# Organ-dependent phytochemical and pharmacological specialization in the endemic *Adenocarpus decorticans* Boiss.

**DOI:** 10.3389/fphar.2026.1919944

**Published:** 2026-10-08

**Authors:** Kaouthar Elbirgui, Houssam Assioui, Othman El Faqer, Saad Harrizi, Ali Zourif, Oussama Rafik, Raffaele Conte, Norddine Habti, Houda Elalami, Hanane Ennaji, Nadia Iounes, Samir Ibenmoussa

**Affiliations:** 1 Laboratory of Chemistry and Biochemistry, Nutrition, Environment and Health, Faculty of Medicine and Pharmacy of Casablanca, Hassan II University of Casablanca, Casablanca, Morocco; 2 Laboratory of Ecology and Environment, Faculty of Sciences Ben M’Sick, Hassan II University of Casablanca, Casablanca, Morocco; 3 Laboratory of Integrative Biology, Faculty of Sciences Ain Chock, Hassan II University of Casablanca, Casablanca, Morocco; 4 Laboratory of Health and Environment and Biotechnology, Faculty of Sciences Ain Chock, Hassan II University of Casablanca, Casablanca, Morocco; 5 Laboratory of Physical-Chemistry, Materials and Catalysis, Faculty of Sciences Ben M’Sick, Hassan II University of Casablanca, Casablanca, Morocco; 6 Research Institute on Terrestrial Ecosystems (IRET), National Research Council (CNR), Naples, Italy; 7 Laboratory of Hematology, Faculty of Medicine and Pharmacy of Casablanca, Hassan II University of Casablanca, Casablanca, Morocco

**Keywords:** *Adenocarpus decorticans* Boiss., antibacterial, antiproliferative, phytochemical profiling, radical-scavenging, UHPLC-ESI-MS

## Abstract

**Introduction:**

*Adenocarpus decorticans* Boiss. is a western Mediterranean shrub restricted to Morocco and Spain, whose phytochemistry and pharmacology remain unexplored despite ethnomedicinal records reported for other species of the genus. This study reports the first organ-resolved characterisation of the species.

**Methods:**

Hydroethanolic extracts of flowers, leaves and stems were quantified for total phenolic, flavonoid and proanthocyanidin contents and profiled by ultra-high-performance liquid chromatography coupled with electrospray ionisation mass spectrometry in targeted selected-ion monitoring mode (UHPLC-ESI-MS/SIM). Radical-scavenging and reducing reactivity was determined by DPPH, ABTS and phosphomolybdenum assays. Antibacterial activity was assessed by disc diffusion with minimum inhibitory and bactericidal concentrations against four Gram-positive and two Gram-negative strains, and antiproliferative activity against P3X63Ag8.653 murine myeloma cells and normal splenocytes by MTT. Molecular docking, 100 ns molecular dynamics simulations and ADMET profiling were performed on the identified metabolites.

**Results:**

Profiling revealed pronounced organ-dependent specialization: flowers were dominated by rosmarinic acid, leaves by flavonol and flavanone glycosides, and stems by procyanidin B2 and diverse flavones. Leaves showed the highest phenolic content (526.90 mg gallic acid equivalents/g extract) and the strongest radical-scavenging reactivity (DPPH IC_50_ 18.62 μg/mL); flowers were the most active against the Gram-positive strains, with bactericidal activity at MIC 0.78–2.08 mg/mL; and stems were the most antiproliferative against P3 cells (IC_50_ 342.66 μg/mL) with the highest selectivity index (1.97). Docking and dynamics identified flavonoid glycosides and proanthocyanidins as the most stable binders, the EGFR–amentoflavone complex being the most stable over 100 ns.

**Discussion:**

These findings reveal clear organ-driven chemical and functional specialization in *A. decorticans*, consistent with the traditional medicinal use documented for the species. Because all assays were performed on crude extracts, the composition-activity correspondences identify candidate constituents rather than establish their contribution, and bioassay-guided fractionation and *in vivo* validation remain necessary.

## 1 Introduction

The emergence and dissemination of antibiotic resistance mechanisms severely compromise the efficacy of standard antimicrobial treatments, impacting public health worldwide (Salam et al., 2023). According to the World Health Organization, this phenomenon could lead to more than 10 million annual deaths by 2050 unless alternative solutions are developed (Talebi Bezmin Abadi et al., 2019). Similarly, cancer remains a dominant factor in global fatalities, with multiple myeloma representing a distinct hematologic malignancy characterized by aberrant plasma cell proliferation within bone marrow microenvironments, which frequently develops resistance to established treatment regimens despite recent therapeutic advances (Garcia-Ortiz et al., 2021).

Oxidative stress constitutes a third critical health concern, serving as a fundamental pathophysiological mediator in numerous chronic conditions, including neoplastic, cardiovascular, and neurodegenerative diseases (Leyane et al., 2022). The convergence of these health challenges, namely antimicrobial resistance, therapeutic limitations in oncology, and oxidative stress-associated pathologies, has catalyzed renewed scientific interest in ethnopharmacological approaches and natural product discovery (McClatchey et al., 2009). Compounds exhibiting multifunctional bioactivities, particularly those demonstrating concurrent antimicrobial, anticancer, and antioxidant properties, merit thorough investigation for their potential to address these complex health issues through complementary mechanisms.

The longstanding ethnobotanical utilization of medicinal plants underscores their potential as a rich source of diverse bioactive molecules, with only a fraction characterized through contemporary scientific methods (Mukti, 2024). Reported estimates suggest that approximately four-fifths of the global population depends primarily on plant-based preparations to satisfy fundamental healthcare requirements (Farnsworth and Soejarto, 1991). The Mediterranean basin, recognized among 36 global biodiversity hotspots, harbors exceptional floristic richness comprising upwards of 25,000 plant species, with approximately 13,000 exhibiting endemism (Mansourian et al., 2013). This extraordinary botanical diversity results from unique convergences of geographic, climatic, and historical influences. Consequently, the Mediterranean region represents a valuable reservoir for novel bioactive compound discovery potentially applicable to antimicrobial resistance, neoplastic diseases, and oxidative stress-related disorders (Oguamanam, 2008).

The strategic geographical positioning of Morocco, situated at the intersection between the Mediterranean, Atlantic, and Saharan ecological zones, has fostered remarkable plant biodiversity encompassing over 4,200 species and subspecies, with 19% demonstrating endemism (Owens, 2004). This botanical wealth has long supported a sophisticated traditional medicine system that utilizes approximately 600 plant species, representing a cultural heritage shaped by Berber, Arab, and Andalusian influences (Laftouhi et al., 2024). Despite this rich ethnobotanical legacy and biodiversity, many endemic Moroccan plants remain scientifically underexplored, with limited investigation into their phytochemical profiles and biological activities (Msanda et al., 2021). This knowledge gap presents a significant opportunity to discover novel bioactive compounds with promising therapeutic applications against the interconnected health challenges of antimicrobial resistance, cancer, and oxidative stress-related disorders.

*Adenocarpus decorticans* Boiss. (Fabaceae) exemplifies such underinvestigated endemic flora. This perennial shrub, distributed across mountainous and semi-arid Moroccan territories, displays characteristic morphological features including trifoliate leaves, vibrant yellow inflorescences, and distinctive exfoliating bark (Velari et al., 2010). The species exhibits remarkable physiological adaptations to adverse environmental conditions such as drought, nutrient-depleted substrates, and intense solar radiation, adaptations potentially facilitated through biosynthesis of specialized secondary metabolites possessing bioactive properties (Yang et al., 2018). Phytochemical investigation of *Adenocarpus* has historically centred on alkaloids. Quinolizidine, pyrrolizidine and bipiperidyl alkaloids are widely distributed across the genus and have long served as chemotaxonomic markers within the Genisteae (Salatino and Gottlieb, 1980; Greinwald et al., 1992). Sparteine-type quinolizidine alkaloids and the pyrrolizidine alkaloid decorticasine have been reported in *A. decorticans*, whereas the ammodendrine-hystrine alkaloids adenocarpine, isoorensine and santiaguine characterise other species, a divergence that has been used to divide the genus into two chemotypic groups (Veen et al., 1992; Essokne et al., 2012). Flavonoid data are more recent and considerably more limited. A chemosystematic survey of 24 species established flavonol and isoflavone glycosides as widespread genus markers (Essokne et al., 2012), and quantitative phenolic determinations have since been reported for *A. bacquei*, *A. anagyrifolius*, *A. artemisiifolius* and *A. complicatus* (Berber et al., 2014; Elgamouz and Bouzekri, 2021; Mechqoq et al., 2022; Benrazzouk et al., 2023). No study has yet resolved the phenolic composition of *A. decorticans* at the organ level or related it to measured biological activity.

As a component of ongoing initiatives to valorize Morocco’s indigenous floristic heritage and identify novel bioactive natural compounds, this investigation presents the first comparative, organ-resolved characterization of *A. decorticans*, integrating phytochemical profiling, multi-assay biological evaluation, and computational analysis across its leaves, stems, and flowers. Hydro-ethanolic extracts from each anatomical structure of Atlas-region specimens were characterized by quantitative determination of phenolic, flavonoid, and proanthocyanidin contents and by UHPLC-ESI-MS/SIM profiling, enabling high-resolution identification of major phenolic metabolites and strengthening structure-activity correlations across plant organs. Antimicrobial efficacy was evaluated against clinically relevant pathogens, including *Micrococcus luteus*, *Staphylococcus epidermidis*, *Staphylococcus aureus*, *Bacillus subtilis*, *Escherichia coli*, and *Pseudomonas aeruginosa*; *in vitro* radical-scavenging and reducing reactivity was assessed through complementary chemical assays (DPPH, ABTS, TAC); and antiproliferative potential was examined against multiple myeloma (P3X63Ag8.653) and splenocyte cells. To provide a mechanistic interpretation of the observed bioactivities at the molecular level, molecular docking, 100 ns molecular dynamics simulations, MM/GBSA binding free-energy analysis, and ADMET profiling were performed on the identified metabolites against relevant antibacterial, anticancer, and antioxidant-related protein targets.

This research contributes to sustainable development initiatives by valorizing Morocco’s indigenous natural resources and addressing critical health challenges. By bridging traditional ethnobotanical knowledge with modern biomedical applications, these findings identify potential bioactive compounds with promising therapeutic properties. The multifunctional nature of these naturally derived substances combining antimicrobial, *in vitro* radical-scavenging, and selective cytotoxic activities, positions them as valuable candidates for innovative therapeutic development aimed at combating antimicrobial resistance, oxidative stress-mediated pathologies, and cancer.

## 2 Materials and methods

### 2.1 Collection and preparation of plant materials

A botanical collection of *A. decorticans* was conducted within the Beni Snassen Forest massif, Tinissane area, Morocco (GPS coordinates: 34°50'26'' N 2°13'16'' W) (Figure 1). The plant was harvested during its flowering stage and taxonomically identified by Taleb Sghir (Scientific Institute of Rabat) as *Adenocarpus decorticans* Boiss. A voucher specimen (RAB 114127) was deposited in the Herbarium of the Scientific Institute of Rabat. After identification, the plant material (leaves, flowers, and stems) was separated, gently washed with distilled water, and air-dried in a shaded, ventilated area for 30 days. The dried material was then ground to a fine powder using a laboratory electric grinder.

**FIGURE 1:**
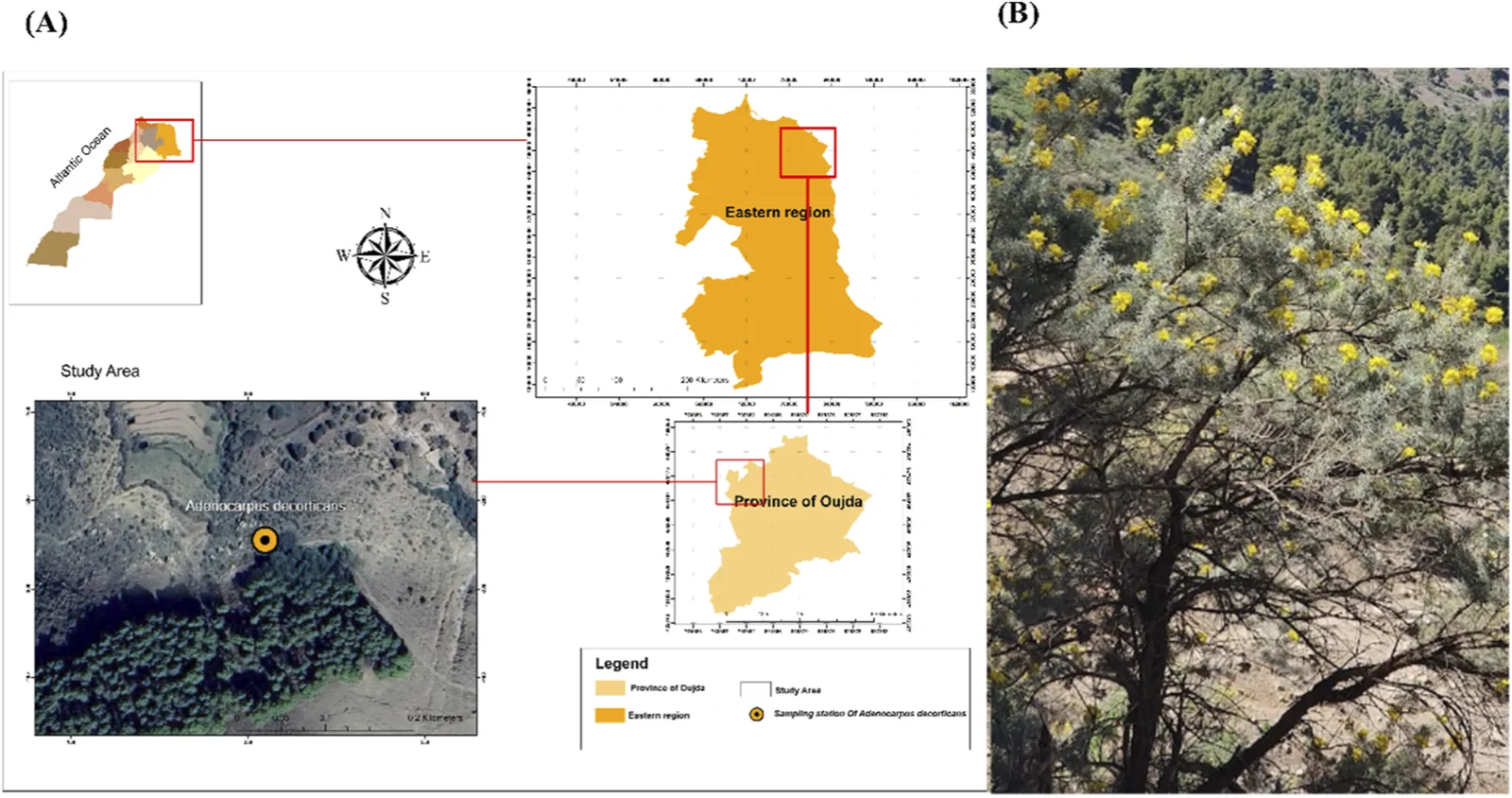
*A. decorticans* Boiss. collection site and morphology. **(A)** Natural habitat and collection in Tinissane area; **(B)** Flowering aerial parts showing characteristic yellow inflorescences.

For extraction, 10 g of powdered material were macerated in 100 mL of an ethanol-water mixture (80:20, v/v) for 72 h at room temperature (20 °C–25 °C) with intermittent agitation. The extract was filtered through Whatman No. 1 filter paper, and the filtrate was concentrated under reduced pressure using a rotary evaporator. Extraction yield was calculated according to Equation 1:

$$\text{Yield }\%=\frac{\text{Mc}\;}{M0}\times 100$$ (1)

Where M_c_ is the mass of the crude extract (g), and M_0_ is the initial mass of plant material (g).

### 2.2 Phytochemical study

#### 2.2.1 Determination of total phenolic compounds (TPC)

The total phenolic compounds (TPC) were quantified using the Folin-Ciocalteu method according to Latif et al*.*, with a slight modification (Latif et al., 2024). A volume of 0.3 mL of each crude extract (125 μg/mL) was mixed with 10% of Folin-Ciocalteu reagent and 7.5% of sodium carbonate solution. The mixture was then incubated in the dark for 2 h. Subsequently, the absorbance was measured at 765 nm using a spectrophotometer UV-63000PC. TPC was calculated using a gallic acid standard curve and expressed in milligrams per gram of gallic acid equivalent (mg GAE/_gE_) and reported as the mean of three replicates with standard deviation.

#### 2.2.2 Determination of total flavonoid compounds (TFC)

The total flavonoid compounds (TFC) were determined according to the method of Ouadghiri et al*.*, with a minor adjustment (Ouadghiri et al., 2026). A 1 mL aliquot of each extract (125 μg/mL) was mixed with 2.4 mL of distilled water and 0.3 mL of a 5% NaNO_3_ solution. After 5 min, 0.3 mL of 10% AlCl_3_ was added, and the mixture was incubated in the dark for 6 min, followed by 1 mL of 1 M NaOH. Finally, the total volume was adjusted to 10 mL with distilled water. The absorbance was immediately measured at 510 nm. The quantification of TFC was performed using a calibration curve expressed in milligrams per gram of quercetin equivalent (mg QE/_gE_), and the results were presented as the mean ± standard deviation of three replicates.

#### 2.2.3 Determination of proanthocyanidin compounds (TPrC)

The total proanthocyanidin compounds (TPrC) were determined according to the method described by Sofidiya et al., with a few adjustments (Sofidiya et al., 2016). A volume of 0.5 mL of each extract at 100 μg/mL was mixed with 3.0 mL of 4% vanillin-methanol solution and 1.5 mL of hydrochloric acid. After a 15-min incubation at room temperature, the absorbance was measured at 500 nm. This test was conducted in triplicate, and the results were expressed as mg CE/_gE_ ± standard deviation.

#### 2.2.4 UHPLC-ESI-MS/SIM analysis of polyphenolic compounds

The phytochemical profiles of the hydroethanolic flower, leaf, and stem extracts of *A. decorticans* were analysed by ultra-high-performance liquid chromatography coupled with electrospray ionisation mass spectrometry operated in targeted selected-ion monitoring mode (UHPLC-ESI-MS/SIM). Analyses were carried out using a Shimadzu Nexera XR LC-40 system coupled to an LCMS-8060 triple-quadrupole mass spectrometer and controlled with LabSolutions software. Before injection, each extract was diluted in water/acetonitrile (1:1, v/v), and 20 μL of this solution were further diluted with 980 μL of acetonitrile. Chromatographic separation was performed on a Kinetex Polar C18 column with a particle size of 2.6 μm using acetonitrile and water containing 0.01% formic acid as the mobile phases. The injection volume was 35 μL, the chromatographic run lasted 25 min, and data were acquired in both positive and negative ionisation modes.

The targeted SIM method included 55 predefined polyphenols and related metabolites. Authentic commercial reference standards were analysed separately during method development under the same chromatographic, ionisation, and source conditions as the extracts. A detected feature was considered standard-matched when its monitored ion and retention time agreed with those established for the corresponding reference standard. Diagnostic product-ion MS/MS spectra and accurate-mass measurements were not acquired. Therefore, when the available SIM and retention-time data did not allow clear discrimination between structural or positional isomers, combined names or tentative annotations were used.

Relative chromatographic abundance was estimated from the integrated peak areas. The %AUC of each feature was calculated as its peak area divided by the summed peak areas of all retained features within the same chromatogram and multiplied by 100. These values represent relative chromatographic responses under the applied SIM conditions and should not be interpreted as absolute metabolite concentrations.

### 2.3 *In vitro* radical-scavenging and reducing reactivity

#### 2.3.1 DPPH radical scavenging assay

The antioxidant capacity of *A. decorticans* extracts was assessed by measuring their ability to scavenge DPPH radical, using the method described by Bendiar et al*.* with slight modifications (Bendiar et al., 2022). A 60 µM solution of DPPH^•^ in ethanol was prepared, 1950 µL aliquots of this solution were mixed with one 50 µL aliquot of the plant extract prepared in ethanol at concentrations ranging from 2.5 to 31.25 μg/mL, ascorbic acid served as a reference standard, and all measurements were performed in triplicate. The reaction mixture was mixed thoroughly and kept in the dark at room temperature for 30 min, after incubation, absorbance was quantified spectrophotometrically at 517 nm. Radical scavenging activity toward DPPH was calculated using Equation 2:

$$\text{DPPH Inhibition }\left(\%\right)=\frac{\text{Abs}\;\text{control}-\text{Abs}\;\text{sample}}{\text{Abs}\;\text{control}}\times 100$$ (2)

Abs Control refers to the absorbance of the blank solution, and Abs Sample corresponds to the absorbance of the sample solution.

#### 2.3.2 ABTS radical scavenging assay

The ABTS assay was performed following the method of Elkoraichi et al., with a slight modification (Elkoraichi et al., 2022). Stock solutions of 7.4 mM ABTS^•+^ and 2.6 mM potassium persulfate were prepared. The working solution was formulated by mixing equal volumes of the two stock solutions and allowing them to react for 16 h in the dark. The resulting working solution was then diluted with ethanol 1:50 (v/v). 10 μL of plant extract at concentrations ranging from 1.25 to 15 μg/mL was mixed with 990 µL of ABTS^•+^ solution and incubated for 6 min. Gallic acid was used as a positive control, and all experiments were conducted in triplicate. The absorbance of the resulting solution was measured spectrophotometrically at 734 nm. The IC_50_ value, which represents the concentration of the sample required to achieve 50% inhibition of the ABTS radical cation, was determined using Equation 3:

$$\text{ABTS Damping }\left(\%\right)=\frac{\text{Abs}\;\text{control}-\text{Abs}\;\text{sample}}{\text{Abs}\;\text{control}}\times 100$$ (3)

Abs Control refers to the absorbance of the blank solution, and Abs Sample corresponds to the absorbance of the sample solution.

#### 2.3.3 Determination of total antioxidant capacity (TAC)

Total antioxidant capacity (TAC) was determined using the phosphomolybdenum method as previously described by Samiry et al. (Samiry et al., 2022). In brief, 100 μL of sample solution at various concentrations was mixed with 900 μL of reagent solution (0.6 M sulfuric acid, 28 mM sodium phosphate, and 4 mM ammonium molybdate). A blank solution was prepared by mixing 100 µL of deionized water with the reagent solution. The tubes were then incubated in a water bath at 95 °C for 90 min. The absorbance was measured using a spectrophotometer at 695 nm. All measurements were replicated three times, and the antioxidant capacity of the samples was quantified using the ascorbic acid equivalent (AAE) assay (Aparadh et al., 2012).

### 2.4 Antibacterial activity

Extract sterility was verified before assay by plating 100 µL of each undiluted extract onto Mueller-Hinton agar and Sabouraud dextrose agar, incubated for 48 h at 37 °C and 25 °C respectively. No bacterial or fungal growth was observed.

#### 2.4.1 Bacterial strains

The antibacterial activities of *A. decorticans* extracts were evaluated against six bacterial strains. The bacterial strains, including *S. aureus* (ATCC 6538), *S. epidermidis* (ATCC 12228), *M. luteus* (ATCC 9341), *E. coli* (ATCC 8739), *B. subtilis* (ATCC 6633), and *P. aeruginosa* (ATCC 27853), were cultured and provided by the Microbiology Unit, Department of Biology, Faculty of Sciences Ben M’Sick, Hassan II University of Casablanca.

#### 2.4.2 Inoculum preparation

Test microorganisms were aseptically streaked onto sterile Mueller-Hinton Agar plates and incubated at 37 °C for 24 h. Six well-isolated colonies were selected from Mueller-Hinton agar cultures. Each colony was inoculated into 5 mL of sterile physiological saline using a sterile wire loop. The bacterial suspensions were vortexed and adjusted to a 0.5 McFarland turbidity standard.

#### 2.4.3 Zone of inhibition test

The zone of inhibition was determined using the disc diffusion method, as described by (Salma et al., 2020). Dried extracts were reconstituted in DMSO at 1 g/mL. Chlortetracycline (3000 μg/mL, 10 µL per disc, corresponding to 30 µg per disc) served as the reference antibiotic, and discs impregnated with DMSO alone served as the negative control; the latter produced no inhibition zone against any of the strains tested. Small bacterial suspensions were aseptically streaked onto MHA plates using a sterile cotton swab. The plates were streaked multiple times, rotating approximately 60° each time, to ensure uniform distribution of the inoculum The antibacterial activity of the crude aqueous-ethanolic extract (1 g/mL) derived from various parts of *A. decorticans* was assessed against two Gram-negative (G-) and four Gram-positive (G+) bacterial strains and sterile filter paper discs (6 mm diameter) were impregnated with 10 µL of the extract and allowed to dry before being placed on the inoculated agar plates. The inoculated plates were incubated upright at 37 °C for 24 h. Following incubation, the diameters of the inhibition zone (DIZ) were measured in mm using a ruler. The activity index (AI) was calculated using Equation 4:

$$\text{AI}=\frac{\text{Diameter}\;\text{of}\;\text{the}\;\text{inhibition}\;\text{zone}\;\text{of}\;\text{sample}}{\text{Diameter}\;\text{of}\;\text{the}\;\text{inhibition}\;\text{zone}\;\text{of}\;\text{antibiotics}}$$ (4)

AI is reported as a descriptive index internal to this screen, not as potency relative to the reference antibiotic, since the extract discs carried 1 g/mL of crude extract against 300 μg/mL on the chlortetracycline discs.

#### 2.4.4 Determination of the minimum inhibitory concentration (MIC)

The minimum inhibitory concentration (MIC) of the plant extract was assessed using a modified version of the method described by Boughroud et al., using 96 well micoplates (Boughroud et al., 2025). Extracts for microdilution were prepared separately in sterile physiological saline, no organic solvent being used at any stage of this assay. 100 μL of Mueller-Hinton broth (MHB) was added to each well of the microplate, followed by 100 µL of plant extract (1 g/mL) to the wells of the first column. The final volume in each well was 100 μL, and the broth containing the extract from the 10th column was then drawn. 40 μL of bacterial suspension was aseptically inoculated into wells 1 through 10 and 12. The plates were then incubated at 37 °C for 24 h.

#### 2.4.5 Determination of minimum bactericidal concentration (MBC)

To determine bacterial growth, contents from wells exhibiting MIC were aseptically subcultured onto antimicrobial-free agar plates, as described in the protocol of Ait Alla et al., and the entire expeiment was performed in triplicate (Alla et al., 2025). In this technique, approximately 3 µL of the contents from each well containing the MIC of the test material, as determined by the microdilution method, were aseptically inoculated onto colony-counting agar plates. The plates were then incubated at 37 °C for 24 h. The lowest concentration of the extract that exhibited no bacterial growth after incubation was identified and recorded as the minimum bactericidal concentration (MBC). Subculture onto antimicrobial-free medium also served to verify that the extracts themselves carried no viable microorganisms, since any organism introduced with the extract would have formed colonies on this drug-free medium. No colony formation was observed at or above the bactericidal endpoints.

### 2.5 Antiproliferative and cytotoxic activity

#### 2.5.1 Experimental animals

Experimental work was performed between March and July 2024 at the Hematology Laboratory of the Faculty of Medicine and Pharmacy, Hassan II University of Casablanca. All animal procedures followed institutional ethical regulations and adhered to the ARRIVE guidelines, under approval reference IRB 00002504 (Ethics declarations). Adult male BALB/c mice were obtained from the institutional Animal House (Faculty of Medicine and Pharmacy, Hassan II University of Casablanca, Morocco). The animals were housed in standard polypropylene cages (five/cage) under controlled environmental conditions (23 °C ± 2 °C, relative humidity 50%–60%, 12 h light/dark cycle) with free access to a standard laboratory diet and water *ad libitum* throughout the study.

#### 2.5.2 Cell line and culture conditions

To evaluate the antiproliferative activity of *A. decorticans* extracts, murine multiple myeloma cell line P3X63Ag8.653 was obtained from the Laboratory of Genetic and Cellular Engineering, Faculty of Medicine and Pharmacy, Casablanca, Morocco. Cells were cultured under conventional laboratory conditions in RPMI 1640 medium, fortified with 10% FBS as a source of growth factors. The culture medium was enhanced with several additives to optimize cellular function: D-(+) Glucose, Sodium pyruvate, HEPES buffer, Na_2_EDTA, L-glutamine and penicillin and streptomycin sulfate antibiotics for contamination prevention. Incubation was carried out at 37 °C under a humidified 5% CO_2_ atmosphere, maintaining physiologically relevant conditions conducive to cellular proliferation (Souhaili et al., 2008).

#### 2.5.3 Splenic mononuclear cells and culture conditions

To assess the extracts’ cytotoxicity, effector cells in the form of mononuclear cells derived from mouse spleen were utilized. Following the ethical procedures described in Section 2.5.1. Three healthy adult male BALB/c mice (5–6 weeks old, 25–30 g) were first anesthetized with an intraperitoneal injection of ketamine (100 mg/kg), and euthanasia was then completed by cervical dislocation to ensure humane and rapid death. Spleens were aseptically excised and placed in separate sterile Petri dishes. After rinsing, cellular components were liberated from splenic tissue through careful perfusion using 15 mL of RPMI culture medium. The cell preparation underwent erythrocyte elimination via treatment with a specific lysis buffer designed for red blood cells. Subsequently, the cellular preparation was brought to a 10 mL volume with RPMI medium and subjected to centrifugation at 1500 rpm for 10 min. To guarantee cellular purity, this process of centrifugation followed by supernatant removal was conducted three times as described by Zandonai et al. (Zandonai et al., 2010).

#### 2.5.4 Growth kinetics and viability

Cell viability was assessed using the trypan blue exclusion method (Strober, 1997). Serial P3 cell dilutions were performed to obtain final concentrations of 4 × 10^4^ and 2 × 10^4^ cells/mL. For splenocytes, concentrations of 8 × 10^5^ and 4 × 10^5^ cells/mL were utilized. Cells were seeded in 96-well plates (200 μL/well) in triplicate. Cell monitoring and counting were performed daily for 8 days to establish growth kinetics under consistent timepoint measurements.

#### 2.5.5 Preparation of treated cells

To evaluate dose-dependent viability, P3 cells were distributed into 96-well plates (4 × 10^4^ cells per well in 100 µL) and allowed to establish for 48 h to reach stable growth conditions. The cells were then exposed to varying concentrations (125–1500 μg/mL) of each extract formulated in 100 µL RPMI medium for 48 h. For splenocytes cells, a different approach was taken; these were plated at 2 × 10^5^ cells per well, and treatment commenced immediately without prior stabilization. The experimental design incorporated melphalan as a positive reference compound to confirm the methodology’s sensitivity in identifying cellular death. A negative reference group of cells maintained in culture medium without additives was established.

#### 2.5.6 Cell viability via tetrazolium reduction

Cytotoxic activity was evaluated by assessing mitochondrial function via the colorimetric MTT assay (Mosmann, 1983), following 48-h treatment exposure. This method measures converting 3-(4,5-dimethylthiazol-2-yl)-2,5-diphenyltetrazolium bromide by metabolically active cells into purple formazan crystals during a 4 h incubation period. After solubilization with DMSO, the resulting-colored solution was analyzed spectrophotometrically at 492 nm, with readings proportionally corresponding to the number of viable cells present (Mouslim et al., 2019). The quantification of cell mortality was performed as a percentage calculation using Equation 5:

$$\text{Cell mortality }\left(\%\right)=100-\frac{\text{Absorbance}\;\text{of}\;\text{treated}\;\text{cells}-\text{Blank}\;\text{absorbance}}{\text{Absorbance}\;\text{of}\;\text{untreated}\;\text{cells}-\text{Blank}\;\text{absorbance}}\;\times \;100$$ (5)

#### 2.5.7 Selectivity index

The selectivity index was calculated as shown in Equation 6 for each organ extract as the ratio between the IC_50_ determined on normal murine splenocytes and the IC_50_ determined on P3 myeloma cells, values above 2 being taken to indicate preferential cytotoxicity toward the tumour cell line and values below 1 indicating greater sensitivity of the normal cells (Suffness et al., 1990).

$$\text{SI}=\frac{{IC}_{50}\;splenocytes\;}{{IC}_{50}\;P3X63Ag8}$$ (6)

### 2.6 *In silico* analysis

#### 2.6.1 Molecular docking study

Molecular docking analysis was realized according to the methods described by El Faqer et al*.*, with slight modifications (El Faqer et al., 2026).

##### 2.6.1.1 Ligand preparation

The three-dimensional structures of the phytochemicals found by UHPLC-ESI-MS/SIM analysis were obtained from the PubChem database (https://pubchem.ncbi.nlm.nih.gov/) with an SDF file extension. In the ligand preparation, the number of molecules from flowers, leaves, and stems were 14, 16, and 19, respectively. These ligand molecules were all converted to PDBQT format using Open Babel v3.1.1 software (O'Boyle et al., 2011), with geometry optimization achieved using the Universal Force Field (UFF) method. Gasteiger partial charges were added, allowing the definition of the number of rotatable bonds to ensure conformational flexibility throughout the docking simulation process.

##### 2.6.1.2 Target protein selection and preparation

The protein targets showing antibacterial, anticancer, and antioxidant properties were selected based on their relevance to drug discovery and the biological activity of the *A. decorticans* extracts. The antibacterial proteins identified included: Enoyl-ACP reductase (FabI; PDB: 1C14 code), DNA gyrase B (PDB: 6F86 code), and sortase A (PDB: 2KID code). The proteins related to anticancer compounds included: Epidermal growth factor receptor (EGFR; PDB: 1M17 code), B-cell lymphoma-2 (Bcl-2; PDB: 4MAN code), and caspase-3 (PDB: 1CP3 code). Finally, the proteins showing antioxidant properties included: Tyrosinase (PDB: 2Y9X). Protein structures were generated with BIOVIA Discovery Studio Visualizer v21.1.0 (Dassault Systèmes, 2021). The water molecules and ligands were deleted, hydrogen atoms were added, and non-polar hydrogens were combined. Kollman united-atom partial charges were assigned before transformation into PDBQT files with AutoDockTools v1.5.7 (Morris et al., 2009).

These targets were selected to represent the principal molecular mechanisms plausibly underlying each measured activity class rather than to mirror a single assay endpoint. FabI, DNA gyrase B and sortase A are canonical antibacterial targets governing fatty-acid biosynthesis, DNA replication and adhesion/virulence, respectively. For antiproliferative activity, Bcl-2 and caspase-3 correspond directly to the apoptotic pathway probed by the MTT assay, while EGFR was included as an EGF-family signalling target implicated in multiple myeloma cell growth (Mahtouk et al., 2005) and a frequent target of the prioritised flavonoids. Tyrosinase, a copper-dependent oxidoreductase and archetypal target of polyphenolic antioxidants, was included to probe the enzymatic redox dimension of antioxidant action, complementing the non-enzymatic radical-scavenging assays (DPPH, ABTS). Docking against this panel was used to generate mechanistic hypotheses rather than to assert defined mechanisms.

##### 2.6.1.3 Molecular docking protocol

Molecular docking studies were carried outusing AutoDock Vina v1.2.0 (Trott and Olson, 2010). The size of the grid box was set to 40 × 40 × 40 Å, along with a grid point size of 0.375 Å, with the origin of the box placed upon the active site of each protein. The active site coordinates of each protein were determined according to the location of co-crystallized compounds or binding residues according to the literature. The value of exhaustiveness was set to 8, while the binding poses included a total of nine poses per protein-ligand combination. The pose with the lowest binding affinity, which corresponds to the highest ΔG binding energy value expressed in kcal/mol, was chosen.

##### 2.6.1.4 Docking protocol validation

The docking protocol was validated by redocking. For each target bearing a drug-like co-crystallised ligand, the native ligand was extracted from its crystal structure and re-docked into the prepared receptor under the same grid, exhaustiveness and scoring settings applied to the phytochemicals, with the search box centred on the crystallographic ligand. Protocol reliability was assessed from the root-mean-square deviation (RMSD) between the top-scoring redocked pose and the crystallographic pose; an RMSD ≤2.0 Å was taken to confirm reproduction of the experimental binding mode.

##### 2.6.1.5 Data analysis and visualization

The binding affinities were classified on the basis of defined cut-offs: ≤ −10.0 kcal/mol, representing very strong affinities; −8.0 to −10.0 kcal/mol, representing strong affinities; −6.0 to −8.0 kcal/mol, representing moderate affinities; and > −6.0 kcal/mol, representing weak affinities. The output docking studies were visualized using BIOVIA Discovery Studio Visualizer for interaction studies, while comparative heat maps were produced using Python v3.11 with the help of Matplotlib and Seaborn libraries.

#### 2.6.2 Molecular dynamics simulation

Molecular dynamics (MD) simulations were performed to evaluate the microscopic stability, structural flexibility, and dynamic behavior of the protein and protein-ligand complexes. All simulations were conducted using GROMACS 2025.1 (Abraham et al., 2015). The protein was parameterized with the CHARMM36 force field, while ligand topologies were generated using SwissParam (Zoete et al., 2011). Prior to simulation, the systems were subjected to energy minimization using the steepest descent algorithm for 2500 steps to remove steric clashes and optimize geometry. Each system was solvated using the SPC water model in a cubic box with periodic boundary conditions. Counter-ions (Na^+^ and Cl^−^) were added using gmx genion to ensure overall charge neutrality.

Following minimization, a two-stage equilibration was performed. First, a 100 ps NVT equilibration was carried out using the v-rescale thermostat to stabilize the temperature at 310 K. This was followed by a 100 ps NPT equilibration using the Parrinello-Rahman barostat to maintain pressure at 1 bar and achieve density equilibration. Position restraints were applied to protein heavy atoms during equilibration. Bond constraints were imposed using the LINCS algorithm, and long-range electrostatic interactions were treated with the Particle Mesh Ewald (PME) method. After equilibration, two unrestrained 100 ns production MD simulation were performed for all systems. Trajectories were saved at appropriate intervals for subsequent analyses.

##### 2.6.2.1 Root mean square deviation (RMSD)

RMSD is a metric that quantifies the distance between frames. It is determined for every profile frame. The root mean square deviation of frame x is calculated.

$${RMSD}_{X}=\sqrt{\frac{1}{N}\sum_{i=1}^{N}{\left(r_{i}^{\prime }\left(t_{x}\right)\right)-r_{i}\left(t_{ref}\right)}^{2}}$$

Where N is the number of selected atoms, r_i_ (t _ref_) represents the position of atom I in the reference structures denotes the position of the same atom in frame x after optimal structural superposition onto the reference. All RMSD calculations were performed using the gmx function in GROMACS 2025.1 and the resulting trajectories were visualized using XmGrace.

##### 2.6.2.2 Root mean square fluctuation (RMSF)

RMSF values can be used to determine the local flexibility based on residue displacements during the MD simulation. The Root Mean Square Fluctuation (RMSF) is useful for characterizing local changes along the protein chain. The RMSF for residue i is calculated using:

$${RMSF}_{I}=\sqrt{\frac{1}{T}\sum_{I=1}^{T}{\left(r_{i}\left(t\right)-\left(r_{i}\right)\right)}^{2}}$$

Where r_i_ (t) represents the position I at time t and r_i_ is its time averaged position. Unlike RMSD, which compares each frame to a reference structure, RMSF characterizes local fluctuations around the mean trajectory. Peaks in the RMSF profile correspond to flexible regions-typically loops and the N or C terminal segments, while lower values indicate more rigid, structurally stable regions of the protein.

##### 2.6.2.3 Radius of gyration (Rg)

The radius of gyration (Rg) represents the mass-weighted root mean square distance of a collection of atoms from their common center of mass. Rg is widely used to evaluate the global compactness and folding stability of biomolecular systems during MD simulations. A stable Rg profile indicates maintenance of tertiary structure, whereas significant deviations imply expansion, unfolding, or structural reorganization. In this study, Rg was calculated for each trajectory frame using GROMACS analysis tools to monitor the compactness of the protein and protein-ligand complexes over the 100 ns simulation.

##### 2.6.2.4 Solvent accessible surface area (SASA)

The Solvent Accessible Surface Area (SASA) was computed using the gmx sasa module in GROMACS 2025.1 along the 100 ns MD trajectory. SASA provides insights into the extent of solvent exposure and potential structural rearrangements at the protein surface. A default probe radius of 0.14 nm was applied, corresponding to the radius of a water molecule. SASA values were extracted at regular intervals for the entire protein-ligand complex to evaluate dynamic changes in surface exposure, folding stability, and solvent interactions throughout the simulation.

##### 2.6.2.5 Hydrogen bond analysis

Hydrogen bond formation and stability were assessed using the gmx hbond module in GROMACS 2025.1 over the 100 ns simulation. Hydrogen bonds were identified using standard geometric criteria: a donor-acceptor distance ≤0.35 nm and a donor-hydrogen-acceptor angle ≥120°. Hydrogen bond counts were calculated for each frame of the trajectory to examine intramolecular stability and to evaluate how ligand binding influenced the persistence and distribution of hydrogen bonds within the protein structure.

#### 2.6.3 ADMET profiling

*In silico* ADMET profiling was performed for all 29 phytochemicals identified by UHPLC-ESI-MS using SwissADME (Daina et al., 2017) Isomeric SMILES strings (Supplementary Table S1) were used as input. Molecular weight (MW), consensus LogP, HBD, HBA, TPSA, gastrointestinal absorption (GIA), BBB permeability, P-glycoprotein substrate probability, CYP3A4 inhibitory potential, and aqueous solubility (LogS; ESOL model) were predicted for each compound (Delaney, 2004). Drug-likeness was assessed against Lipinski’s Rule of Five (Lipinski et al., 1997) and four additional filters (Ghose et al., 1999; Veber et al., 2002; Egan et al., 2000); a composite bioavailability score (F20%) was calculated as the fraction of the five filters satisfied. Brenk structural alerts were evaluated for all compounds. All ADMET parameters reported here are computationally predicted values and are interpreted throughout as predictive estimates requiring experimental validation.

##### 2.6.3.1 Toxicity prediction

Computational toxicity profiling was performed using pkCSM (Pires et al., 2015). AMES mutagenicity, hepatotoxicity, hERG cardiotoxicity (hERG I and II), oral rat acute toxicity (LD_50_), and skin sensitisation were predicted for all 29 compounds.

##### 2.6.3.2 Multi-filter drug-likeness compliance and bioavailability score

Compliance with five drug-likeness frameworks was assessed simultaneously and a composite bioavailability score (F20%) was calculated as the fraction of filters satisfied (range 0.00–1.00) (Ghose et al., 1999; Veber et al., 2002; Egan et al., 2000).

##### 2.6.3.3 Target prediction and structural similarity to approved drugs

Human protein target prediction for the four prioritized leads (amentoflavone, kaempferol, rosmarinic acid, ursolic acid) was performedusing SwissTargetPrediction with *Homo sapiens* as the target organism (Daina and Zoete, 2019). Targets with predicted probability (Ω) ≥ 0.30 were retained. Tanimoto structural similarity to approved drugs was computed using RDKit (Landrum, 2023).

##### 2.6.3.4 PASS biological activity prediction

Biological activity spectra for the four lead compounds were predicted using PASS Online (Pogodin et al., 2018) with *H. sapiens* as the target organism. SMILES strings (Supplementary Table S1) were used as input. Activities with Pa >0.70 and Pa > Pi were retained and classified into pharmacological categories: antimicrobial, anticancer/apoptosis, kinase/signalling, antioxidant/redox, cardioprotective/vascular, and metabolic/hepatoprotective. Predicted adverse effects were extracted separately.

### 2.7 Statistical analysis

All biological assays were performed in three independent experiments (n = 3), with each condition measured in technical triplicate. Technical replicates were averaged to obtain one value per independent experiment and were not treated as separate observations. Results are expressed as mean ± standard deviation. Normality of the ANOVA residuals and homogeneity of variance were assessed using the Shapiro-Wilk and Brown-Forsythe tests, respectively. Differences among groups were analysed using one-way ANOVA followed by Tukey’s multiple-comparison test, with *p < 0.05* considered statistically significant, performed with GraphPad Prism 8.0.2 software (GraphPad Software Inc.). PCA was performed on centred and standardised data as an exploratory multivariate analysis using the Rstudio software for Windows, version 2026.01.0 + 392 (RStudio Team, 2024).

## 3 Results

### 3.1 Phytochemical analysis

#### 3.1.1 Extraction yield and quantitative phytochemical screening

In this work, hydroethanolic extracts of *A. decorticans* were subjected to qualitative and quantitative phytochemical screening. The percentage of extractable material obtained through hydroethanolic extraction from the various anatomical parts of *A. decorticans* showed considerable differences, with stems giving the lowest yield (9.3%), followed by flowers (12.96%), and leaves the highest (17.94%).

TPC, TFC, and TPrC contents of *A. decorticans* hydroethanolic extracts are summarized in Table 1. TPC showed significant organ-dependent variation, with leaves exhibiting the highest levels (526.90 ± 11.60 mg GAE/_gE_), followed by flowers (314.69 ± 2.61 mg GAE/_g gE_) and stems (258.77 ± 1.35 mg GAE/_gE_) (*p < 0.05*). TFC followed the same trend, with leaves again being the richest fraction (369.23 ± 7.14 mg QE/_gE_), followed by flowers (190.76 ± 4.22 mg QE/_gE_) and stems (174.02 ± 3.46 mg QE/_gE_) (*p < 0.05*). In contrast, TPrC was highest in stems (31.64 ± 0.62 mg CE/_gE_), with lower contents in leaves (26.15 ± 2.10 mg CE/_gE_) and flowers (18.38 ± 1.70 mg CE/_gE_).

**TABLE 1:** Total phenolic, flavonoids and proanthocyanidin content of *A. decorticans* hydroethanolic extracts.

Plant part	TPC (mg GAE/_gE_)	TFC (mg QE/_gE_)	TPrC (mg CE/_gE_)
Flowers	314.69 ± 2.61^a^	190.76 ± 4.22^a^	18.38 ± 1.7^a^
Leaves	526.90 ± 11.61^b^	369.23 ± 7.14^b^	26.15 ± 2.10^b^
Stems	258.77 ± 1.35^c^	174.02 ± 3.46^c^	31.64 ± 0.62^c^

Results are presented as mean ± SD (n = 3). Within a row, values with different superscript letters differ significantly (*p < 0.05*).

#### 3.1.2 UHPLC-ESI -MS analysis

A detailed UHPLC-ESI-MS profiling was performed on *A. decorticans* extracts derived from stems, leaves, and flowers, with the corresponding results presented in Table 2. UHPLC-ESI-MS analysis revealed pronounced organ-dependent differences in the phytochemical profiles of the flowers, leaves, and stems of *A. decorticans*, particularly in terms of chemical diversity and dominant metabolite classes.

**TABLE 2:** UHPLC-MS profile of the *A. decorticans* flowers, leaves, and stems extracts.

Plant part	Compound	Formula	Class	RT (min)	Monitored ion (m/z)	AUC	% AUC
Flowers	Caffeic acid	C_9_H_8_O_4_	Hydroxycinnamic acid	1.506	179.0 [M−H]−	9,591,756	0.84%
	trans-Ferulic acid	C_10_H_10_O_4_	Hydroxycinnamic acid	1.909	193.0 [M−H]−	136,250,088	11.96%
	Rosmarinic acid	C_18_H_16_O_8_	Hydroxycinnamic acid derivative	2.005	359.0 [M−H]−	918,480,011	80.60%
	Quercetin	C_15_H_10_O_7_	Flavonol	2.522	301.0 [M−H]−	26,500,009	2.33%
	Syringic acid	C_9_H_10_O_5_	Hydroxybenzoic acid	5.736	198.9 [M + H]+	48,770,533	4.28%
Leaves	Caffeic acid	C_9_H_8_O_4_	Hydroxycinnamic acid	1.483	179.0 [M−H]−	5,592,888	0.17%
	trans-Ferulic acid	C_10_H_10_O_4_	Hydroxycinnamic acid	2.097	193.0 [M−H]−	21,047,257	0.65%
	Epicatechin	C_15_H_14_O_6_	Flavan-3-ol	2.340	289.0 [M−H]−	19,345,184	0.59%
	Rutin	C_27_H_30_O_16_	Flavonol diglycoside	2.484	609.0 [M−H]−	360,577,712	11.09%
	Kaempferol-3-O-rutinoside	C_27_H_30_O_15_	Flavonol diglycoside	2.654	593.1 [M−H]−	210,241,473	6.47%
	Quercetin-3-O-glucoside	C_21_H_20_O_12_	Flavonol glycoside	2.656	463.1 [M−H]−	245,972,481	7.57%
	Isorhamnetin-7-O-pentoside	C_21_H_20_O_11_	Methylated flavonol glycoside	3.172	447.1 [M−H]−	356,219,373	10.96%
	p-Coumaric acid	C_9_H_8_O_3_	Hydroxycinnamic acid	3.253	162.9 [M−H]−	1,683,197	0.05%
	Myricetin	C_15_H_10_O_8_	Flavonol	4.110	317.0 [M−H]−	703,031,503	21.62%
	Trimethoxyflavone	C_18_H_16_O_5_	Methoxylated flavone	9.022	312.0 [M]−	56,136,814	1.73%
	Procyanidin B2	C_30_H_26_O_12_	Proanthocyanidin	11.067	577.0 [M−H]−	651,319,417	20.03%
	Kaempferol-3-O-arabinoside	C_20_H_18_O_10_	Flavonol glycoside	11.408	417.1 [M−H]−	266,565	0.01%
	Naringin	C_27_H_32_O_14_	Flavanone glycoside	12.056	579.0 [M−H]−	380,609,490	11.71%
	Quercetin-3-O-glucuronide	C_21_H_18_O_13_	Flavonol glucuronide	12.759	477.0 [M−H]−	103,773,026	3.19%
	Arbutin	C_12_H_16_O_7_	Hydroquinone glycoside	14.726	271.2 [M−H]−	135,521,956	4.17%
Stems	Caffeic acid	C_9_H_8_O_4_	Hydroxycinnamic acid	1.449	179.0 [M−H]−	4,551,464	0.13%
	trans-Ferulic acid	C_10_H_10_O_4_	Hydroxycinnamic acid	2.170	193.0 [M−H]−	6,635,975	0.19%
	Kaempferol-3-O-rutinoside	C_27_H_30_O_15_	Flavonol diglycoside	2.398	593.1 [M−H]−	162,904,092	4.78%
	Quercetin-3-O-rutinoside	C_27_H_30_O_16_	Flavonol diglycoside	2.425	609.1 [M−H]−	45,223,577	1.33%
	Epigallocatechin	C_15_H_14_O_7_	Flavan-3-ol	2.446	305.0 [M−H]−	49,499,638	1.45%
	Isorhamnetin-7-O-pentoside	C_21_H_20_O_11_	Methylated flavonol glycoside	2.641	447.1 [M−H]−	531,565,974	15.58%
	Quercetin-3-O-glucoside	C_21_H_20_O_12_	Flavonol glycoside	2.675	463.1 [M−H]−	215,479,496	6.32%
	p-Coumaric acid	C_9_H_8_O_3_	Hydroxycinnamic acid	3.265	162.9 [M−H]−	6,097,854	0.18%
	Kaempferol-3-O-glucuronide	C_21_H_18_O_12_	Flavonol glucuronide	3.355	461.1 [M−H]−	60,654,792	1.78%
	Luteolin	C_15_H_10_O_6_	Flavone	5.752	284.9 [M−H]−	309,917,534	9.09%
	Kaempferol	C_15_H_10_O_6_	Flavonol	5.761	285.0 [M−H]−	472,357,793	13.85%
	Apigenin	C_15_H_10_O_5_	Flavone	6.938	269.0 [M−H]−	194,285,336	5.70%
	Trimethoxyflavone	C_18_H_16_O_5_	Methoxylated flavone	9.359	312.0 [M]−	29,654,856	0.87%
​	Chlorogenic acid	C_16_H_18_O_9_	Hydroxycinnamic acid ester	9.600	353.0 [M−H]−	31,715,114	0.93%
	Procyanidin B2	C_30_H_26_O_12_	Proanthocyanidin	11.043	577.0 [M−H]−	622,196,862	18.24%
	Naringin	C_27_H_32_O_14_	Flavanone glycoside	12.185	579.0 [M−H]−	220,599,676	6.47%
	Amentoflavone	C_30_H_18_O_10_	Biflavonoid	12.650	537.1 [M−H]−	36,699,856	1.08%
	Quercetin-3-O-glucuronide	C_21_H_18_O_13_	Flavonol glucuronide	12.888	477.0 [M−H]−	129,835,410	3.81%
	Arbutin	C_12_H_16_O_7_	Hydroquinone glycoside	14.711	271.2 [M−H]−	236,714,903	6.94%
	Ursolic acid	C_30_H_48_O_3_	Pentacyclic triterpenoid	15.676	455.0 [M−H]−	44,473,392	1.30%

The flower extract had the simplest profile, with five detected compounds, and was largely dominated by rosmarinic acid, which accounted for 80.60% of the total AUC. trans-Ferulic acid was the second most abundant constituent at 11.96%, whereas syringic acid, quercetin, and caffeic acid were present at lower levels. The leaf extract was more diverse and mainly characterized by myricetin (21.62%) and procyanidin B2 (20.03%). Naringin, rutin, and isorhamnetin-7-O-pentoside were also abundant, representing 11.71%, 11.09%, and 10.96%, respectively, followed by quercetin-3-O-glucoside and kaempferol-3-O-rutinoside. The stem extract showed the greatest chemical diversity, with procyanidin B2 as the main compound (18.24%), followed by isorhamnetin-7-O-pentoside (15.58%), kaempferol (13.85%), and luteolin (9.09%). Arbutin, naringin, quercetin-3-O-glucoside, and apigenin were also detected at appreciable levels. Overall, the flowers were mainly characterized by hydroxycinnamic acid derivatives, whereas the leaves and stems contained a broader range of flavonoids, flavonoid glycosides, and proanthocyanidins. These profiles indicate functional compartmentalisation rather than a gradient of phenolic abundance, with each organ accumulating the metabolite classes matched to its physiological role.

### 3.2 Evaluation of *in vitro* radical-scavenging and reducing reactivity

The *in vitro* radical-scavenging and reducing reactivity of *A. decorticans* extracts was evaluated using three complementary spectrophotometric assays: DPPH, ABTS, and TAC, and the results are summarized in Figure 2; Table 3. The hydroethanolic extracts of *A. decorticans* showed dose and organ-dependent antioxidant activity (Figure 2), with the leaf extract displaying the strongest radical-scavenging performance, as indicated by the lowest DPPH IC_50_ (18.62 ± 0.83 μg/mL) and ABTS IC_50_ (4.41 ± 0.06 μg/mL), together with the highest TAC (296.43 ± 11.01 AAE/_gDW_) (Table 3). In contrast, the flower and stem extracts exhibited weaker scavenging activities, with comparable DPPH IC_50_ values (21.56 ± 0.61 and 21.79 ± 0.99 μg/mL, respectively; *p > 0.05*) but significantly different ABTS IC_50_ values (8.50 ± 0.19 vs. 9.90 ± 0.11 μg/mL; *p < 0.05*), and markedly lower TAC in stems (170.87 ± 8.42 AAE/_gDW_) compared with flowers (266.15 ± 7.13 AAE/_gDW_) (*p < 0.05*) (Table 3).

**FIGURE 2:**
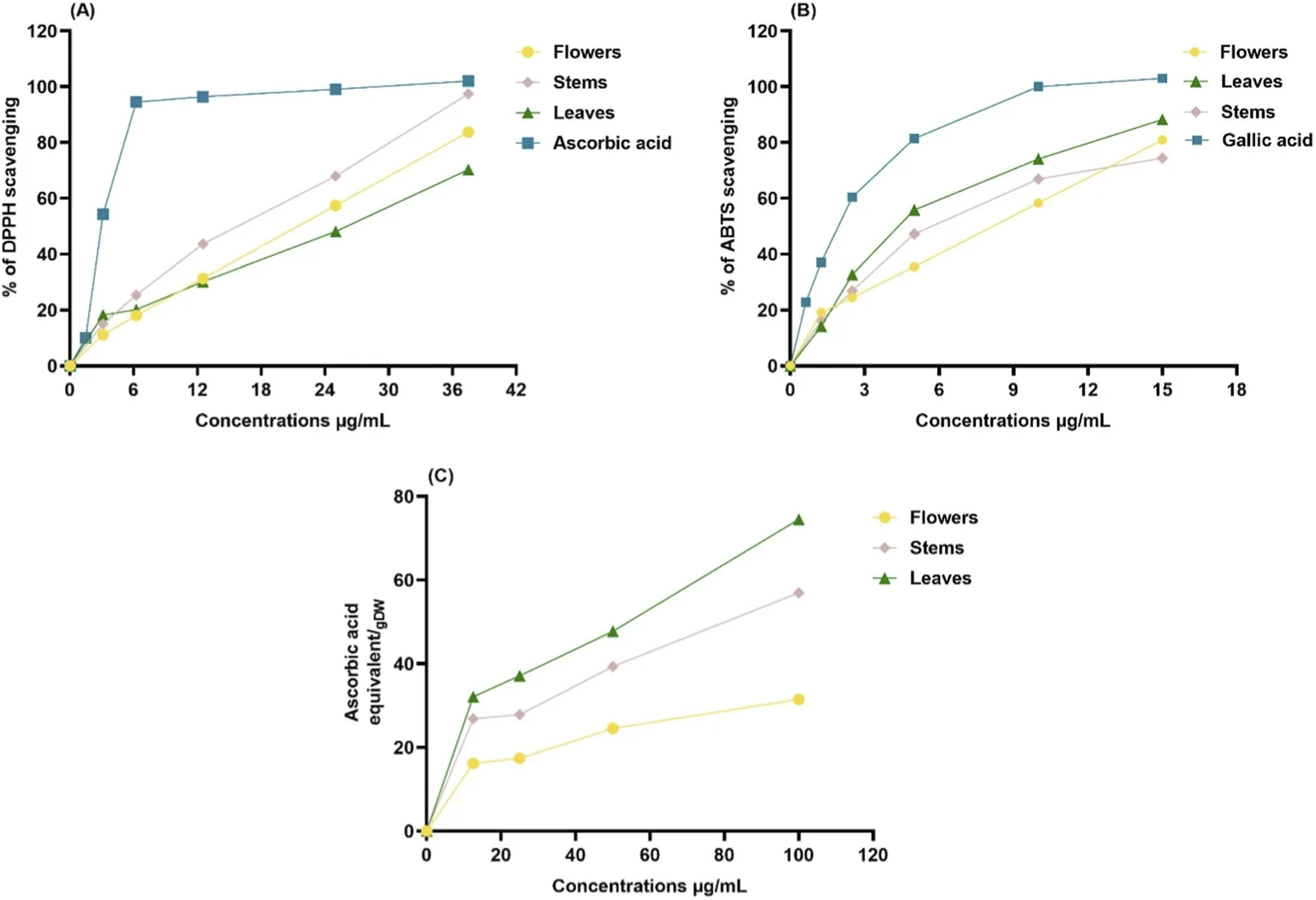
Antioxidant activity of *A. decorticans* hydroethanolic extracts, expressed as the percentage scavenging of DPPH^•^ **(A)** and ABTS^•+^ **(B)**, and as ascorbic acid equivalents for TAC **(C)**.

**TABLE 3:** Antioxidant activity of *A. decorticans* hydroethanolic extracts.

Sample	DPPH IC_50_ (µg/mL)	ABTS IC_50_ (µg/mL)	TAC AAE/_gDW_
Flowers	21.56 ± 0.61^a,ns^	8.50 ± 0.19^a^	266.15 ± 7.13^a^
Leaves	18.62 ± 0.83^b^	4.41 ± 0.06^b^	296.43 ± 11.01^b^
Stems	21.79 ± 0.99^c,ns^	9.90 ± 0.11^c^	170.87 ± 8.42^c^
Ascorbic acid	2.82 ± 0.04	-	-
Gallic acid	-	1.90 ± 0.11	-

Results are presented as mean ± SD (n = 3). Within the same column, means assigned different superscript letters (a, b, and c) are significantly different (*p* < 0.05), whereas “ns” indicates no significant difference (p > 0.05).

### 3.3 Evaluation of antibacterial activity

The antibacterial activity of hydroethanolic extracts obtained from the flowers, leaves, and stems of *A. decorticans* was quantified by MIC and MBC and by the derived MBC/MIC ratios, which define the bactericidal or bacteriostatic mode of action (Table 4). DIZ and AI, was used beforehand as a preliminary qualitative screen to prioritise the most responsive organs and strains for quantitative testing (Table 5; Figure 3).

**TABLE 4:** Determination of MIC and MBC of *A. decorticans* hydroethanolic extracts.

		Bacterial strains			
Plant part	Parameter	*S. aureus*	*S. epidermidis*	*M. luteus*	*E. coli*
Flowers	MIC (mg/mL)	0.78	2.08	1.04	20.83
	MBC (mg/mL)	1.04	5.20	1.56	>500
	MBC/MIC ratio	1.33	2.5	1.50	24
	Effect	Bactericidal	Bactericidal	Bactericidal	Bacteriostatic
Leaves	MIC (mg/mL)	8.33	16.66	1.30	41.66
	MBC (mg/mL)	10.41	33.33	2.60	>500
	MBC/MIC ratio	1.24	2.006	2.00	12.001
	Effect	Bactericidal	Bactericidal	Bactericidal	Bacteriostatic
Stems	MIC (mg/mL)	12.50	3.33	20.83	200
	MBC (mg/mL)	20.83	83.33	33.33	>500
	MBC/MIC ratio	1.66	25.02	1.60	2.5
	Effect	Bactericidal	Bacteriostatic	Bactericidal	Bacteriostatic

**TABLE 5:** Determination of the diameter of inhibition zones (mm) of *A. decorticans* hydroethanolic extracts.

	Bacterial strains					
Sample	*S. aureus*	*S. epidermidis*	*P. aeruginosa*	*E. coli*	*M. luteus*	*B. subtilis*
Flowers	36.33 ± 1.11^a^	37.66 ± 0.44^a^	-	25.66 ± 0.44^a^	39.00 ± 0.66^a^	-
Leaves	27.66 ± 0.88^a^	28.50 ± 0.33^a^	-	14.16 ± 0.77^a^	32.00 ± 0.66^a^	-
Stems	20.00 ± 0.66^a^	17.33 ± 0.44^a^	-	-	19.66 ± 0.44^a^	-
Chlortetracycline	36.66 ± 1.11	36.00 ± 1.33	21.00 ± 0.38	18.66 ± 0.44	36.00 ± 1.33	22.00 ± 0.93

Results are presented as mean ± SD (n = 3). Within the same column, means assigned different superscript letters are significantly different (*p* < 0.05), whereas means sharing the same superscript letter are not significantly different (*p* > 0.05). “-” indicates no detectable inhibition zone. Inhibition-zone diameters are expressed in millimetres (mm) and include the 6 mm diameter of the paper disc.

**FIGURE 3:**
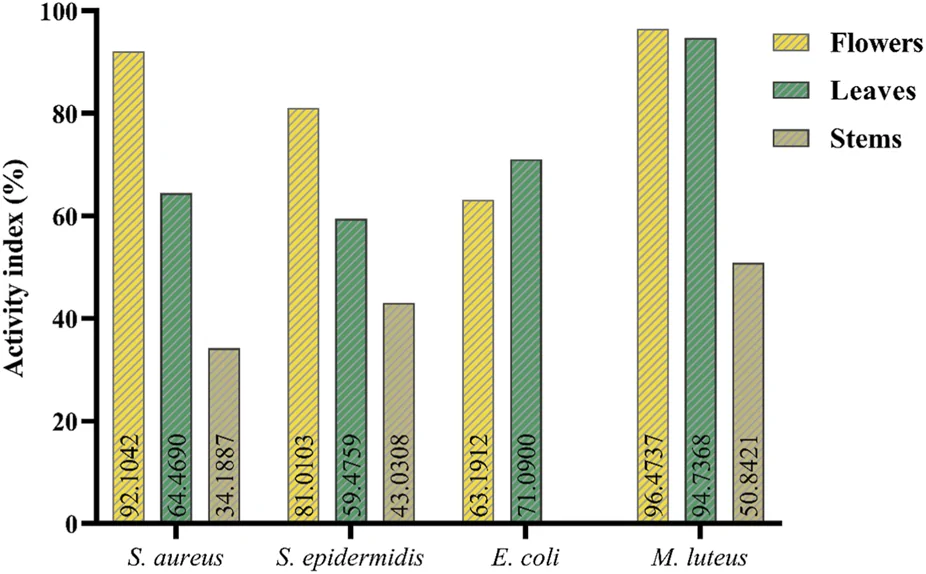
Activity index of *A. decorticans* hydroethanolic extracts.

Among the three organs, the flower extract was the most active against the Gram-positive strains. It showed MIC values of 0.78 mg/mL for *S. aureus*, 1.04 mg/mL for *M. luteus*, and 2.08 mg/mL for *S. epidermidis*, corresponding to moderate antibacterial activity for a crude, unfractionated extract, and the associated MBC/MIC ratios below four established a bactericidal mode of action against all three strains (Pankey and Sabath, 2004). This quantitative activity was consistent with the preliminary screen, in which the flower extract produced the largest inhibition zones against *M. luteus* (39.00 ± 0.66 mm), *S. epidermidis* (37.66 ± 0.44 mm), and *S. aureus* (36.33 ± 1.11 mm), with AI values exceeding 80%. In contrast, activity against the Gram-negative *E. coli* was markedly weaker, with a higher MIC of 20.83 mg/mL and no detectable MBC at concentrations up to 500 mg/mL, indicating a bacteriostatic effect against this strain.

The leaf extract was less active than the flower extract but retained bactericidal activity against the Gram-positive strains. Its MIC values were 1.30 mg/mL for *M. luteus*, 8.33 mg/mL for *S. aureus*, and 16.66 mg/mL for *S. epidermidis*, and the corresponding MBC/MIC ratios indicated a bactericidal mode of action against all tested Gram-positive bacteria, whereas the effect against *E. coli*, with a MIC of 41.66 mg/mL, was bacteriostatic. In the preliminary screen these extracts gave intermediate inhibition zones, ranging from 14.16 ± 0.77 mm for *E. coli* to 32.00 ± 0.66 mm for *M. luteus*, with AI values between 59% and 95%.

The stem extract was the least active of the three organs, with substantially higher MIC and MBC values across all strains. *S. epidermidis* showed the lowest MIC (3.33 mg/mL), although the elevated MBC/MIC ratio of 25.02 indicates a bacteriostatic effect, whereas *M. luteus* was the only strain against which the stem extract was bactericidal (MIC 20.83 mg/mL, MBC 33.33 mg/mL), whereas the activity against *S. epidermidis* was bacteriostatic, as reflected by a markedly elevated MBC/MIC ratio of 25.02. Activity against *E. coli* was minimal, with growth inhibition only at a MIC of 200 mg/mL and no measurable MBC above 500 mg/mL, indicating a bacteriostatic and pharmacologically negligible effect at the tested concentrations. In the preliminary screen the stem extract accordingly produced the smallest inhibition zones and the lowest AI values.

Taken together, the MIC and MBC data establish a clear organ-dependent, bactericidal, and Gram-positive-selective antibacterial profile, with a potency ranking of flowers > leaves > stems and a consistently greater susceptibility of Gram-positive strains than of the Gram-negative *E. coli*. Because these values were obtained with crude, unfractionated extracts in which the active constituents are diluted within a complex phenolic matrix, they are expected to underestimate the potency of the individual bioactive compounds and identify bioassay-guided fractionation as the priority next step.

### 3.4 Evaluation of cytotoxic activity

#### 3.4.1 P3 cell viability kinetics

The proliferation kinetics of P3 cells were monitored over a 9-day period at two initial seeding densities (2 × 10^4^ and 4 × 10^4^ cells/mL; Figure 4). At both densities, cultures exhibited a short lag phase during the first 1–2 days, followed by a marked exponential growth phase extending up to day 4. Cells seeded at 4 × 10^4^ cells/mL reached a higher maximal density (2.8 × 10^6^ cells/mL) than those seeded at 2 × 10^4^ cells/mL, which peaked at 1.7 × 10^6^ cells/mL.

**FIGURE 4:**
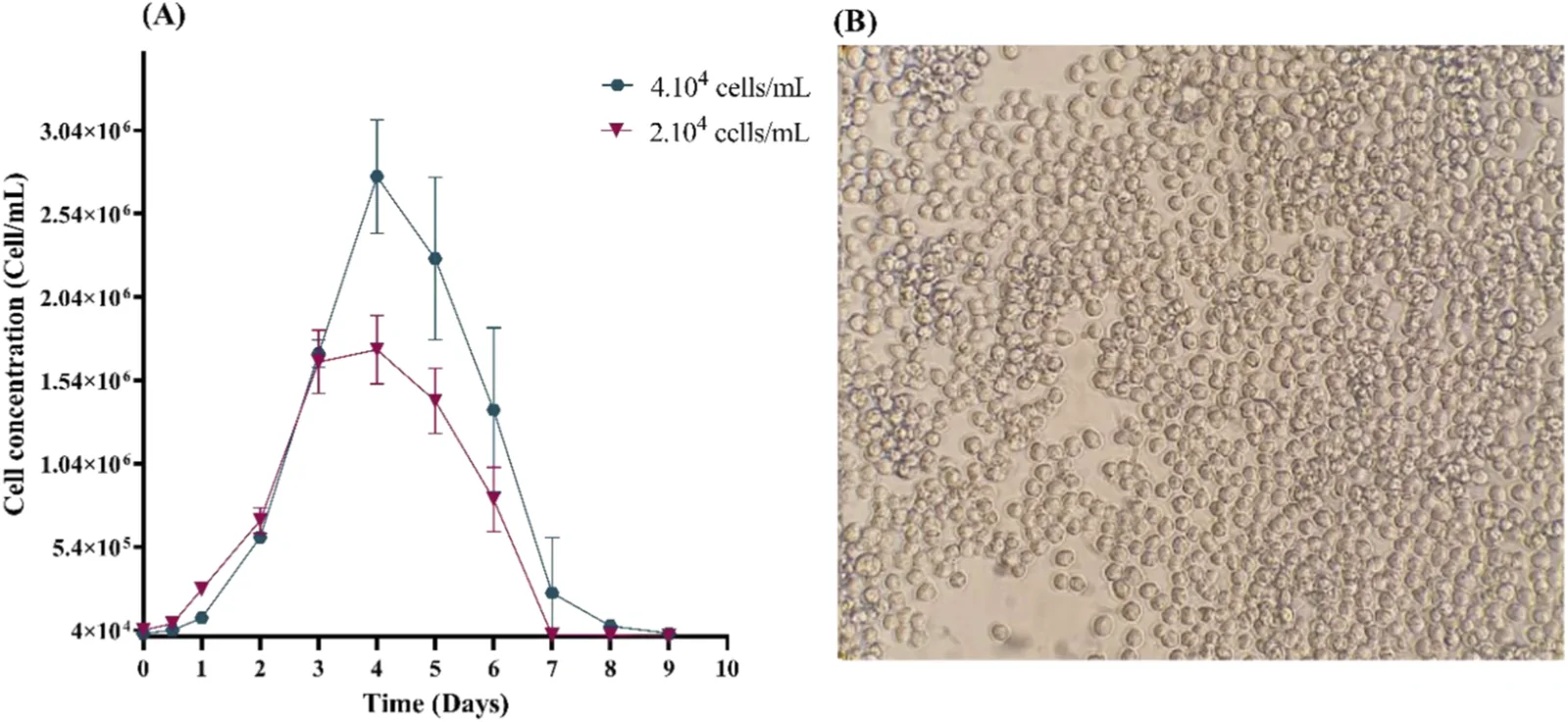
Proliferation kinetics **(A)** and phase-contrast morphology **(B)** of P3 myeloma cells cultured at two initial seeding densities.

After day 4, both cultures entered a decline phase (days 4–7), with a more pronounced loss of viability observed in the higher-density culture. By day 7, both populations approached a terminal phase, and cell viability was nearly abolished by day 9. Despite the two-fold difference in initial seeding density, the cultures displayed parallel growth trajectories throughout the experiment, with the higher-density condition consistently maintaining greater cell numbers during both the proliferative and decline phases.

#### 3.4.2 Splenic mononuclear cells viability

In contrast to P3 cells, splenic mononuclear cells (splenocytes) showed a progressive and continuous decline in viability over time at both seeding densities (Figure 5). Cultures initiated at 8 × 10^5^ cells/mL exhibited a steeper decrease in cell numbers, declining from the initial density to nearly undetectable levels within approximately 100 h. Cells seeded at 2 × 10^5^ cells/mL followed a similar trend but with a less pronounced rate of decline, ultimately reaching minimal viable counts at a comparable time point. These results indicate a limited proliferative capacity and reduced survival of splenocytes under the experimental conditions.

**FIGURE 5:**
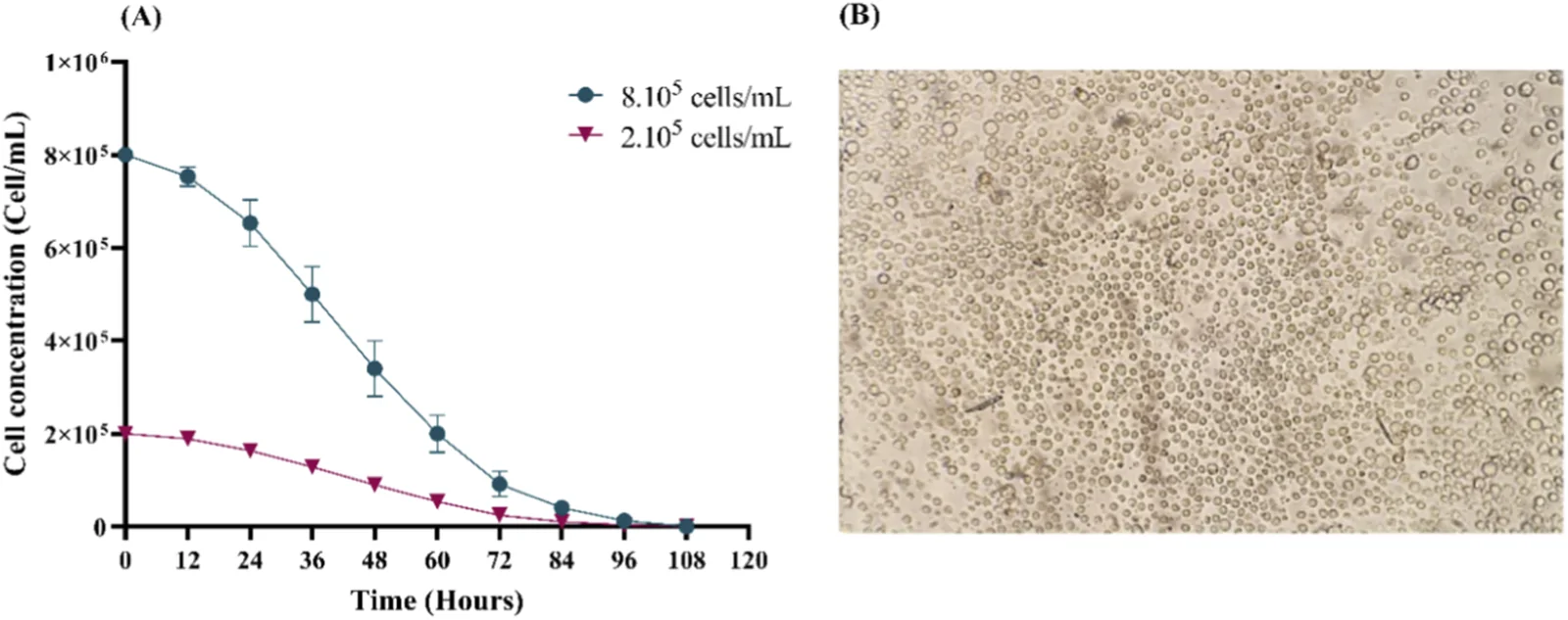
Proliferation kinetics **(A)** and phase-contrast morphology **(B)** of splenocytes cultured at different initial seeding densities.

#### 3.4.3 Dose-dependent cytotoxicity and selectivity

The cytotoxic effects of hydroethanolic extracts derived from the flowers, leaves, and stems of *A. decorticans* were assessed against murine P3 myeloma cells and splenocytes, with the corresponding results presented in Figure 6; Table 6. Across the tested concentration range (125–2000 μg/mL), all extracts produced a dose-dependent decrease in viability in both P3 cells and splenocytes.

**FIGURE 6:**
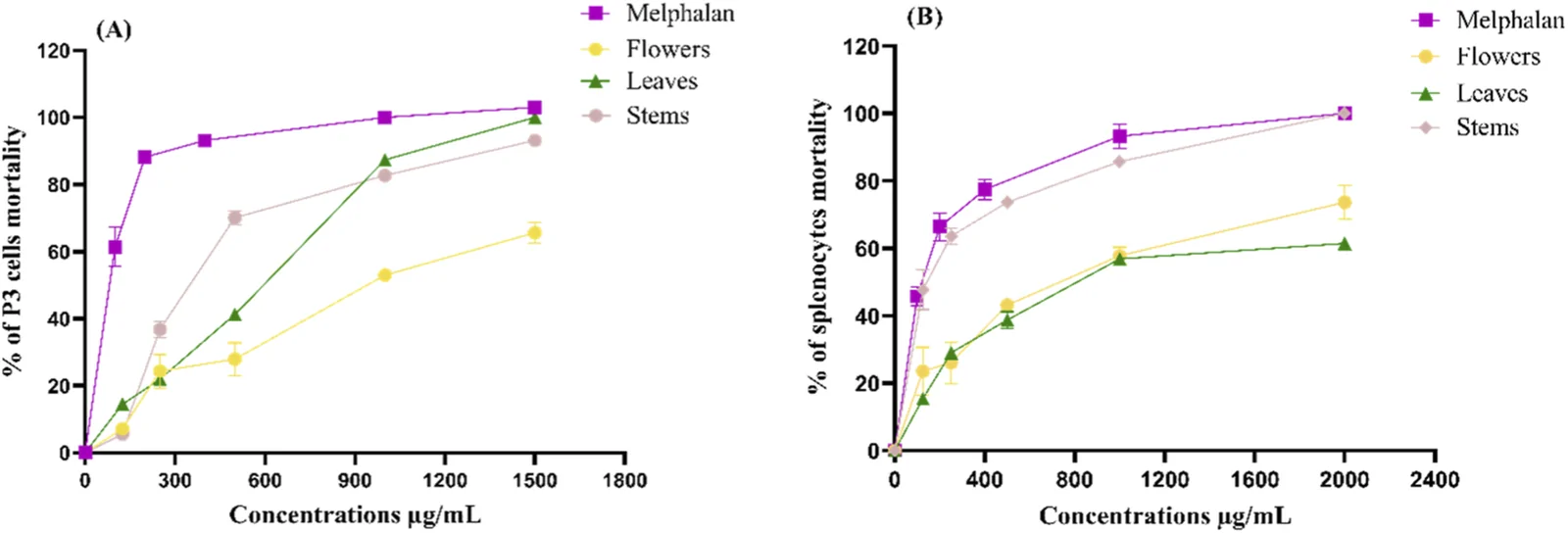
Dose-dependent cytotoxic effects of *A. decorticans* hydroethanolic extracts on P3 myeloma cells **(A)** and splenocytes **(B)**.

**TABLE 6:** Antiproliferative activity of *A. decorticans* hydroethanolic extracts on P3 cancer cell lines and splenocytes, expressed as IC_50_ (µg/mL).

Cell lines	*A. decorticans* extracts			Melphalan
	Flowers	Leaves	Stems	
P3 cell lines	909 ± 23.02^a^	498 ± 10.95^b^	342.66 ± 8.08^c^	200.7 ± 11.93^a,c^
Splenocytes	143.56 ± 14.77^a^	881.5 ± 17.73^b^	675.1 ± 69.2^c^	173.8 ± 27.4^ns^
Selectivity index	0.16	1.77	1.97	0.87

Results are presented as mean ± SD (n = 3). Within the same row, IC_50_ values assigned different superscript letters (a, b, and c) are significantly different (*p* < 0.05), whereas values sharing at least one superscript letter are not significantly different (*p* > 0.05). "ns" indicates no statistically significant difference (*p* > 0.05). The selectivity index (SI) was calculated as the ratio of the IC_50_ value for splenocytes to that for P3 cancer cells (SI = IC_50_ splenocytes / IC_50_ P3 cells).

The cytotoxic response of P3 cells differed significantly according to the plant organ, with the stem extract showing the greatest potency with an IC_50_ of 342.66 ± 8.08 μg/mL, followed by the leaf extract with an IC_50_ of 498 ± 10.95 μg/mL, whereas the flower extract was the least active and required nearly two-fold higher concentrations than stems to achieve 50% inhibition (IC_50_ = 909 ± 23.02 μg/mL) (Table 6). Accordingly, the cytotoxic potency ranked as stems > leaves > flowers, with significant differences in IC_50_ values (*p < 0.05*).

In splenocytes the organ ranking was inverted relative to P3 cells (Table 6). The flower extract was the most cytotoxic toward normal splenocytes, with an IC_50_ of 143.56 ± 14.77 μg/mL, a value below that of melphalan (173.8 ± 27.4 μg/mL), followed by the stem extract (675.1 ± 69.2 μg/mL), while the leaf extract was the least cytotoxic (881.5 ± 17.73 μg/mL). Cytotoxic potency against splenocytes therefore ranked as flowers > melphalan > stems > leaves.

Selectivity indices quantify this preferential activity (Table 6). The flower extract returned an SI of 0.16, being approximately 6.3-fold more cytotoxic to normal splenocytes than to P3 cells. The leaf and stem extracts returned SI values of 1.77 and 1.97 respectively, indicating modest preferential activity toward the malignant line that remains below the conventional threshold of 2 (Suffness et al., 1990), while melphalan returned an SI of 0.87.

### 3.5 *In silico* analysis

#### 3.5.1 Molecular docking analysis

The docking protocol was first validated by redocking the native co-crystallised ligands of four representative targets spanning the antibacterial and anticancer classes. The crystallographic binding modes were accurately reproduced, with top-pose RMSD values of 0.24 Å (FabI, 1C14), 1.16 Å (Bcl-2, 4MAN), 1.15 Å (DNA gyrase B, 6F86) and 1.31 Å (EGFR, 1M17), all within the 2.0 Å threshold, confirming that the protocol reliably reproduces experimental binding modes.

Molecular docking simulations were performed to evaluate the binding potential of phytochemicals identified in *A. decorticans* against selected antibacterial, anticancer, and antioxidant-related protein targets. The docking results highlighted compound-specific and target-specific interaction profiles, revealing strong to very strong binding affinities, particularly among flavonoid glycosides and proanthocyanidins. The results are presented in Figure 7.

**FIGURE 7:**
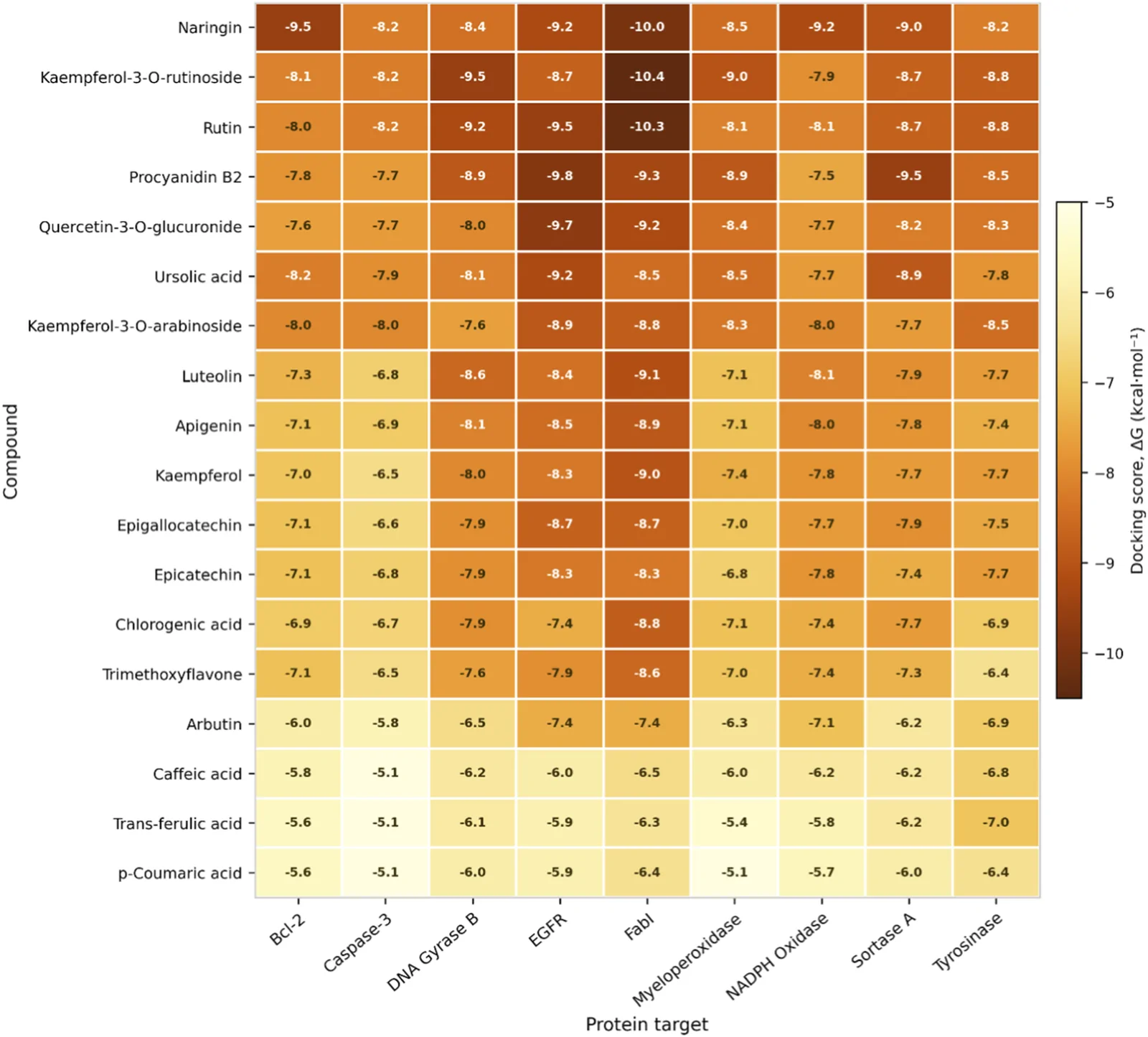
Comparative heatmap of top phytochemicals binding affinities across protein targets in *A. decorticans* flower, leaf, and stem extracts.

Among the antibacterial targets, FabI exhibited high binding affinities with several flavonoid compounds. Procyanidin B2 derivatives, rutin, naringin, and kaempferol derivatives demonstrated strong to very strong interaction energies, suggesting their potential to inhibit bacterial fatty acid biosynthesis. DNA gyrase B also showed favorable binding interactions with glycosylated flavonoids, including rutin, luteolin derivatives, and kaempferol derivatives. Furthermore, sortase A displayed notable affinity toward polyphenolic compounds, particularly procyanidin B2 and structurally related molecules, supporting their possible anti-virulence properties.

Regarding anticancer targets, Bcl-2 exhibited the most favorable binding affinities among the proteins evaluated. Compounds such as procyanidin B2, naringin, rutin, and isorhamnetin derivatives showed strong interactions with Bcl-2, with calculated binding energies approaching or below −9.0 kcal/mol, indicating potential anti-apoptotic pathway modulation. EGFR demonstrated moderate to high binding affinities with several flavonoid glycosides, whereas caspase-3 exhibited comparatively lower interaction energies, suggesting weaker or less direct binding to this apoptotic effector protein.

For antioxidant-related targets, docking results indicated substantial binding affinities for flavonoid glycosides and polyphenolic compounds. Rutin, naringin, kaempferol derivatives, and myricetin demonstrated consistent interaction profiles, supporting their potential role in modulating oxidative stress-related pathways. The reproducibility and stability of the docking scores further reinforce the likelihood of their contribution to radical scavenging and redox regulation mechanisms.

#### 3.5.2 Comparative analysis across compounds

Comparison of the average binding affinity for all targets revealed that flavonoid glycosides, especially rutinosides and glucosides, were consistently in the top binding compounds. Procyanidin B2 showed high binding affinity for antibacterial and anticancer targets, whereas rutin and naringin showed high multi-target binding affinity. These observations support the hypothesis that polyphenolic compounds are the major contributors of the observed bioactivity (Figure 8).

**FIGURE 8:**
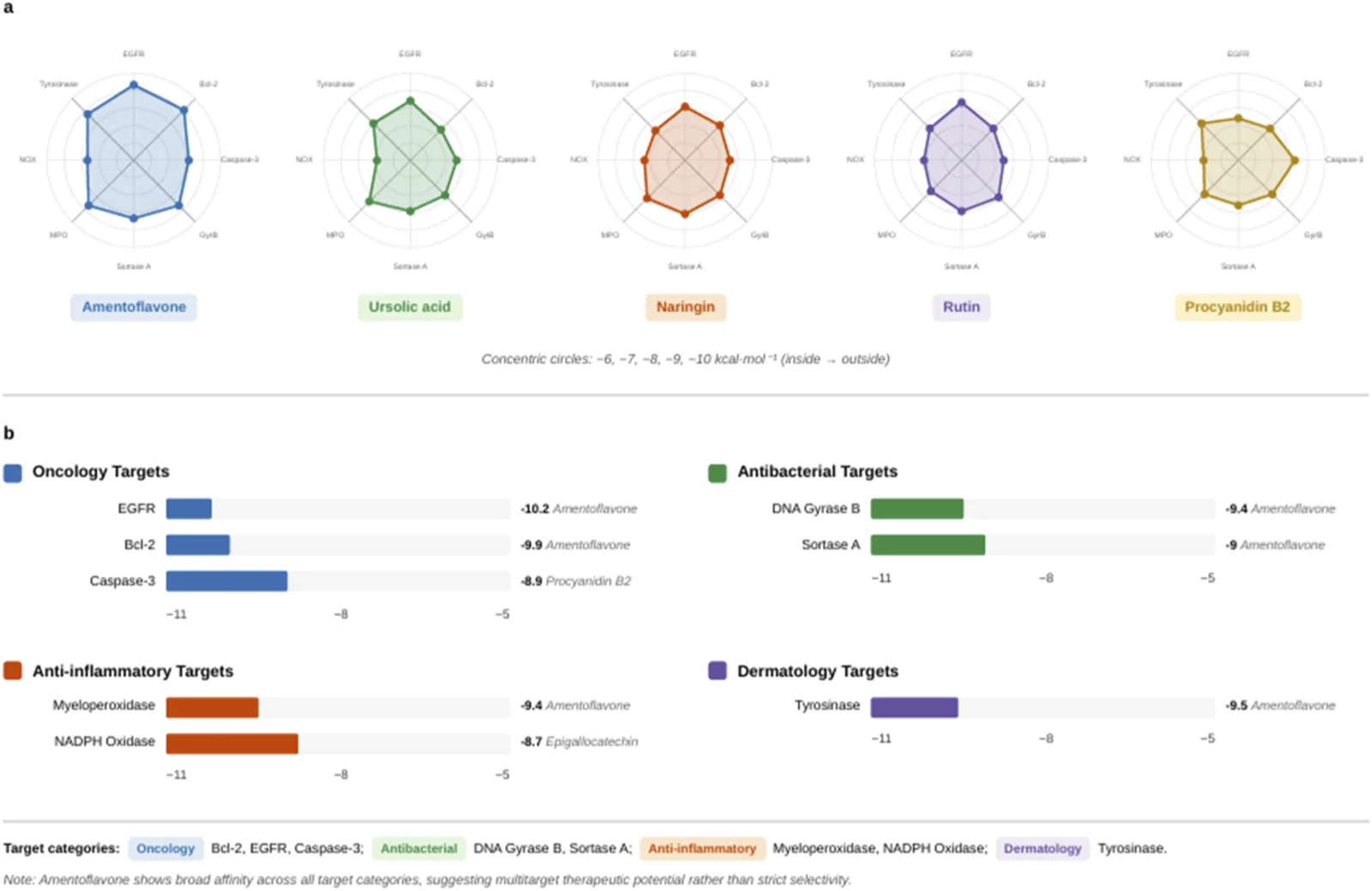
Radar plot illustrating comparative binding profiles of top-performing compounds. **(a)** Individual radar plots comparing binding profiles of the top five compounds across eight protein targets. Distance from center represents binding strength (outer edge = stronger binding, ΔG = −11 kcal·mol^−1^; center = weaker binding, ΔG = −5 kcal·mol^−1^). **(b)** Best-performing compound for each protein target, organized by therapeutic category.

#### 3.5.3 Molecular dynamics simulation

##### 3.5.3.1 Root mean square deviation (RMSD)

The structural stability of the two retained protein-ligand complexes was evaluated by backbone RMSD over the 100 ns production run (Figure 9A). The EGFR-amentoflavone complex equilibrated after approximately 30 ns and fluctuated around 1.0–1.3 nm, whereas the Bcl-2-amentoflavone complex showed larger backbone drift, rising to approximately 2.0 nm during the second half of the trajectory. In both complexes the ligand remained associated with the binding site throughout, with a low ligand RMSD of approximately 0.13–0.18 nm, indicating that amentoflavone did not dissociate despite the moderate backbone mobility of the receptors.

**FIGURE 9:**
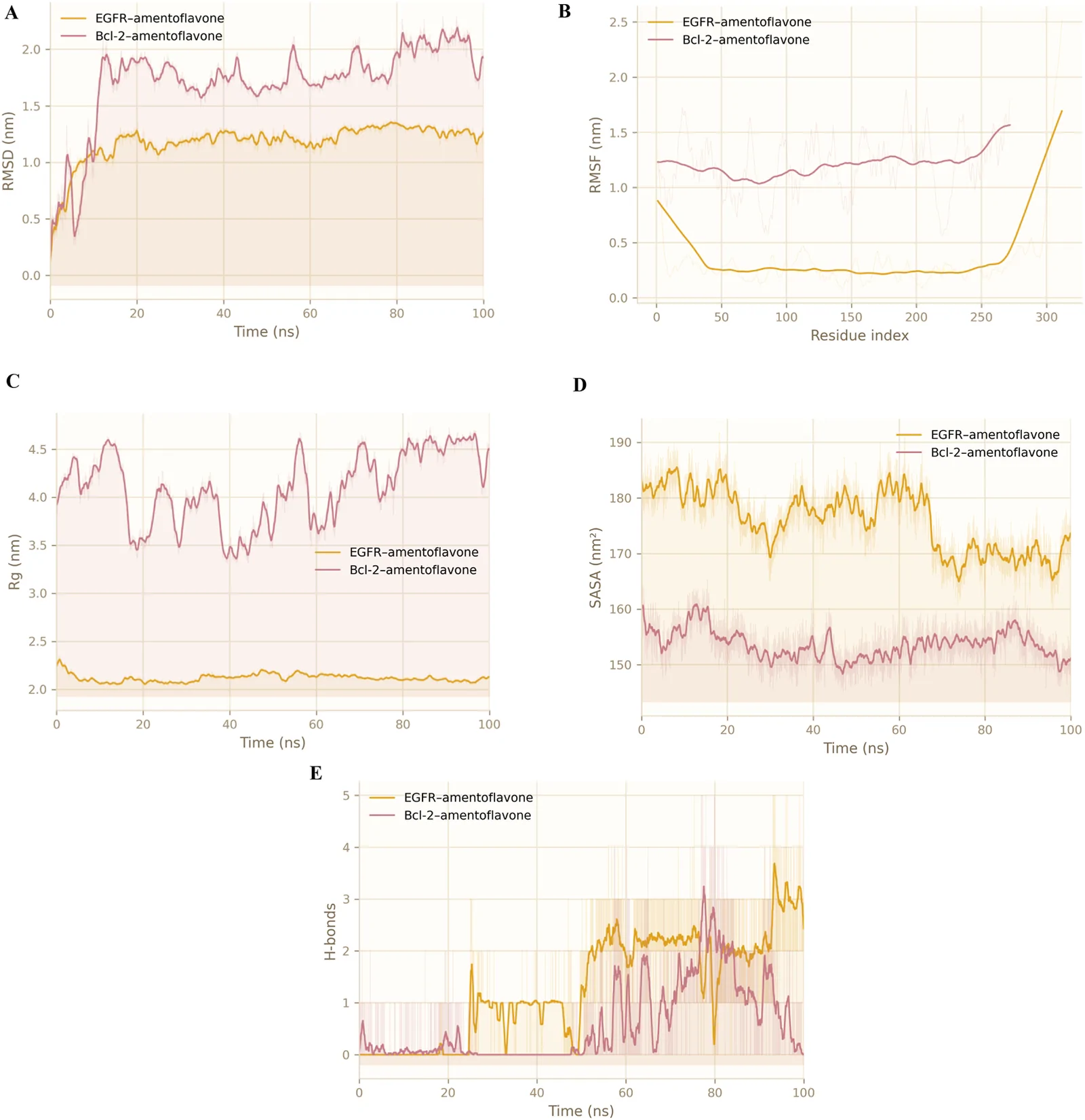
**(A)** RMSD trajectories of the EGFR-amentoflavone (orange) and Bcl-2-amentoflavone (red) complexes during the 100-ns molecular dynamics simulations. **(B)** Per-residue RMSF profiles of the EGFR-amentoflavone (orange) and Bcl-2-amentoflavone (red) complexes. **(C)** Radius of gyration (Rg) trajectories of the EGFR-amentoflavone (orange) and Bcl-2-amentoflavone. (red) complexes. **(D)** Solvent-accessible surface area (SASA) profiles of the EGFR-amentoflavone (orange) and Bcl-2-amentoflavone (red) complexes. **(E)** Time evolution of hydrogen-bond numbers in the EGFR-amentoflavone (orange) and Bcl-2-amentoflavone (red) complexes.

##### 3.5.3.2 Root mean square fluctuation (RMSF)

Residue-level flexibility was assessed through RMSF analysis (Figure 9B). The EGFR-amentoflavone complex showed predominantly moderate fluctuations (mean ≈0.34 nm), with larger excursions confined to loop and terminal regions. The Bcl-2-amentoflavone complex displayed higher overall flexibility (mean ≈1.2 nm), consistent with its larger backbone RMSD and radius of gyration.

##### 3.5.3.3 Radius of gyration (Rg)

The compactness of the complexes was examined through the radius of gyration (Figure 9C). The EGFR-amentoflavone complex maintained a stable Rg of approximately 2.1 nm throughout the trajectory, whereas the Bcl-2-amentoflavone complex retained a larger and mildly increasing Rg of approximately 4.1–4.5 nm, consistent with its greater size and backbone mobility.

##### 3.5.3.4 Solvent accessible surface area (SASA)

Solvent-accessible surface area was analysed to assess solvent exposure and conformational change (Figure 9D). Both complexes maintained stable SASA profiles, fluctuating around 170–185 nm^2^ for the EGFR-amentoflavone complex and 150–165 nm^2^ for the Bcl-2-amentoflavone complex, indicating no large-scale unfolding or solvent-exposure change over the production run.

##### 3.5.3.5 Hydrogen bond analysis

Hydrogen-bond analysis characterised the ligand–protein polar contacts (Figure 9E). The EGFR-amentoflavone complex maintained on average one to three hydrogen bonds, with occasional peaks up to five, whereas the Bcl-2-amentoflavone complex formed fewer and more intermittent hydrogen bonds (mean below one, absent in the majority of frames), indicating weaker and more transient polar interactions.

#### 3.5.4 ADMET profiling

The ADMET descriptors reported in this section are *in silico* predictions generated by computational models (SwissADME, pkCSM); they are presented as hypothesis-generating estimates requiring experimental confirmation and do not represent established pharmacokinetic or toxicological properties of the compounds.

SwissADME-based ADMET profiling of the 29 phytochemicals revealed a clear dichotomy between aglycone/small phenolic acid forms and glycosylated compounds (Supplementary Table S2). Fifteen compounds fully complied with Lipinski’s Ro5: the four leads (amentoflavone, kaempferol, rosmarinic acid, ursolic acid) plus quercetin, myricetin, caffeic acid, ferulic acid, trans-ferulic acid, syringic acid, p-coumaric acid, epicatechin, epigallocatechin, arbutin, and trimethoxyflavone. These compounds displayed favourable physicochemical profiles (TPSA <120 Å^2^, consensus LogP <5) compatible with oral absorption. Ursolic acid was the sole Ro5 violator among the leads (LogP = 5.93), consistent with its triterpenoid skeleton; it was retained for its established pentacyclic bioactivity. The 14 glycosylated compounds systematically exceeded Ro5 thresholds for MW (>500 g/mol) and HBD/HBA, with TPSA values of 140–270 Å^2^ and low predicted GIA as intact molecules, consistent with their established biotransformation by gut microbiota to active aglycone forms. CYP3A4 inhibitory potential was flagged for kaempferol, quercetin, luteolin, apigenin, and trimethoxyflavone. No compound triggered Brenk structural alerts. The ADMET pharmacokinetic profiles of the four leads are summarised in Figure 10.

**FIGURE 10:**
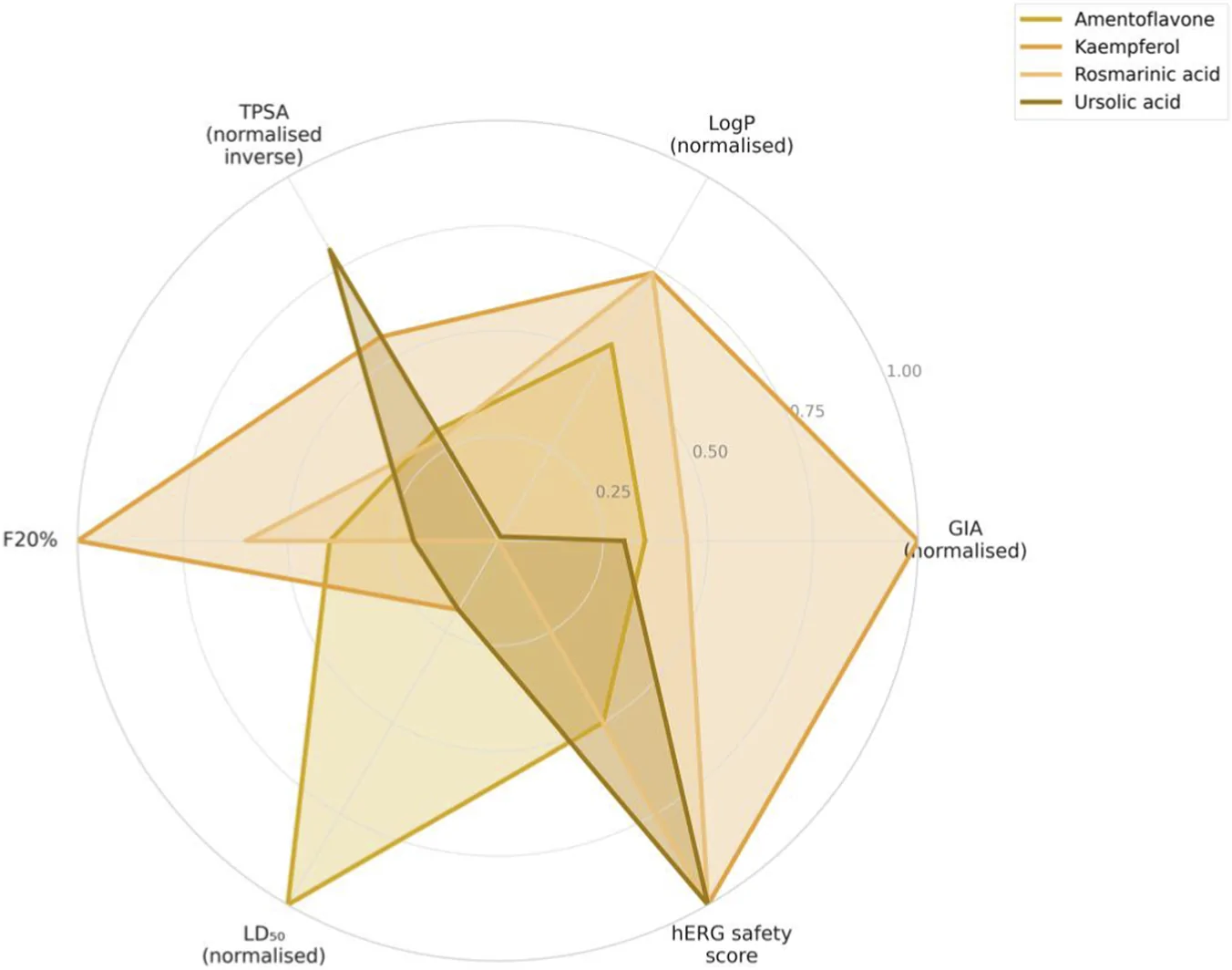
ADMET pharmacokinetic radar chart for the four prioritized lead compounds from *A. decorticans* Boiss. Six normalised pharmacokinetic axes are shown. GIA: gastrointestinal absorption (High = 1.00; Low = 0.30–0.45 proportional to predicted intestinal absorption). LogP: normalised as 1 − |LogP|/6 (higher = more drug-like lipophilicity). TPSA: normalised inverse as 1 − TPSA/200 (higher = better absorption). F20%: composite bioavailability score (fraction of five drug-likeness filters satisfied; range 0.00–1.00). LD_50_: normalised from the observed lead range (2.24–2.82 log mol/kg; higher = less acutely toxic). hERG safety score: 1.00 = no hERG flags; 0.50 = hERG II inhibitor flag only; 0.00 = both hERG I and II flags. Larger polygon area indicates a more favourable overall pharmacokinetic profile. Data from SwissADME (Daina et al., 2017) and pkCSM (Pires et al., 2015).

##### 3.5.4.1 Toxicity prediction

pkCSM-based toxicity profiling revealed a favourable overall safety profile for the prioritized leads (Supplementary Table S3). Amentoflavone, rosmarinic acid, and ursolic acid were predicted non-mutagenic (AMES negative) and non-hepatotoxic. Kaempferol returned a positive AMES prediction, consistent with class-level flavonol pro-oxidant activity at supratherapeutic concentrations. Oral rat LD_50_ values for the four leads ranged from 2.24 to 2.82 log mol/kg (ca. 170–660 mg/kg), consistent with GHS Category 4–5 acute toxicity. hERG I inhibition was not predicted for any lead; hERG II inhibition was flagged for amentoflavone, warranting experimental patch-clamp validation. Ursolic acid was flagged for hepatotoxicity at supratherapeutic concentrations, consistent with established dose-dependent hepatic effects. Rosmarinic acid showed clean safety flags across all endpoints (AMES negative, non-hepatotoxic, hERG negative; LD_50_ = 2.24 log mol/kg). The computational toxicity profile across all compounds is shown in Figure 11B.

**FIGURE 11:**
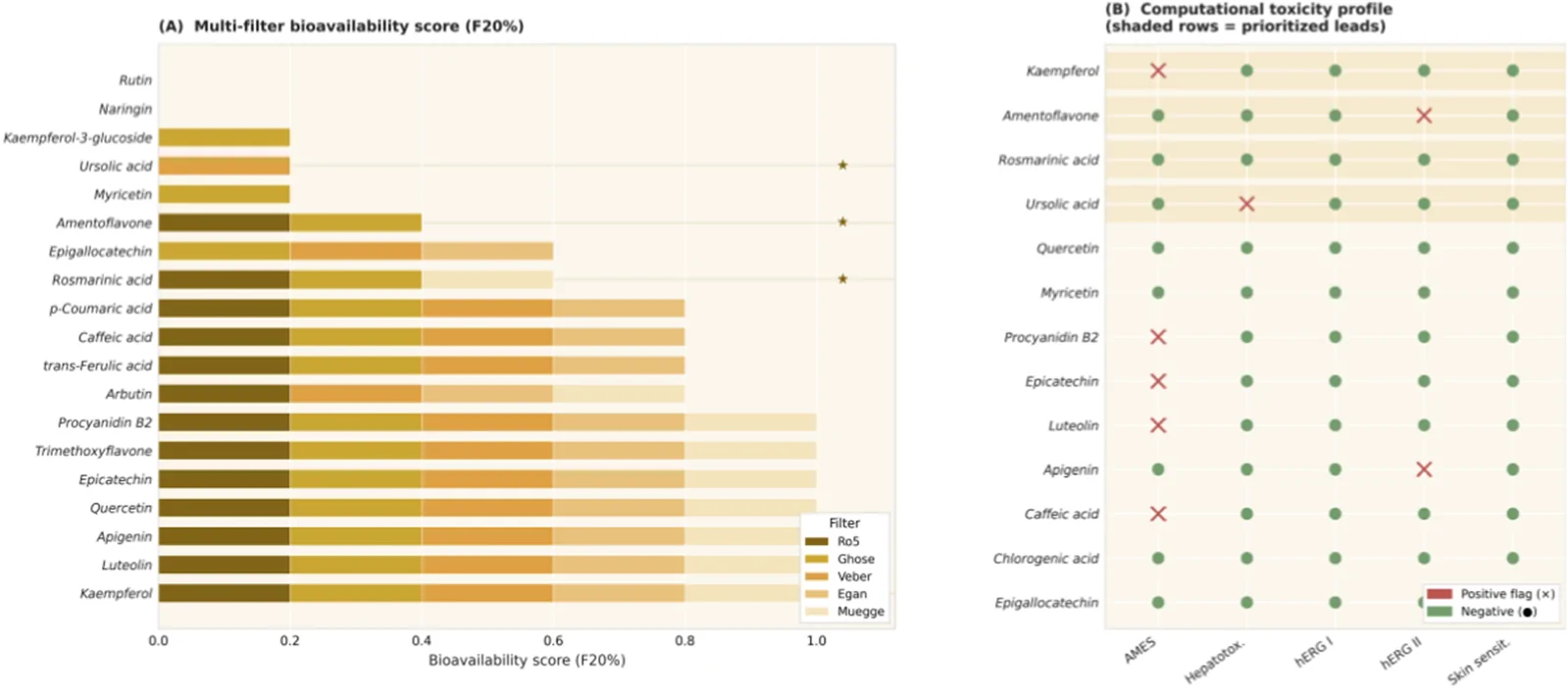
Multi-filter drug-likeness compliance and computational toxicity profiles for phytochemicals identified in *A. decorticans* Boiss. **(A)** Composite bioavailability score (F20%) for all 29 phytochemicals identified by UHPLC-ESI-MS. Each coloured segment represents one drug-likeness filter (Ro5, Ghose, Veber, Egan, Muegge); a filled segment indicates filter compliance (0.20 per filter). Stars (★) denote the four prioritized leads. **(B)** Computational toxicity dot matrix (pkCSM; Pires et al., 2015). Green circles (•) = negative prediction; red crosses (×) = positive flag. Endpoints shown: AMES mutagenicity, hepatotoxicity, hERG I inhibitor, hERG II inhibitor, and skin sensitisation. Shaded rows indicate the four prioritized leads.

##### 3.5.4.2 Multi-filter drug-likeness compliance and bioavailability score

Kaempferol satisfied all five drug-likeness frameworks, yielding the maximum bioavailability score (F20% = 1.00; Supplementary Table S4). Rosmarinic acid satisfied three filters (F20% = 0.60), with Veber and Egan violations attributable to TPSA (144 Å^2^). Amentoflavone satisfied two filters (F20% = 0.40); it was retained as the principal lead given its MD-validated binding to EGFR and Bcl-2. Ursolic acid satisfied only the Veber filter (F20% = 0.20), consistent with its triterpenoid physicochemical profile. Glycosylated compounds scored F20% ≤ 0.20, consistent with their classification as dietary polyphenols. Multi-filter compliance profiles across all 29 compounds are shown in Figure 11A.

##### 3.5.4.3 Target prediction and structural similarity to approved drugs

SwissTargetPrediction identified mechanistically coherent human protein targets for the four leads (Supplementary Table S5). Amentoflavone and kaempferol shared 26 common predicted targets (Ω ≥ 0.50), including NOX4 (Ω = 1.00), AKR1B1 (Ω = 1.00), CDK5 (Ω = 1.00), FLT3 (Ω = 1.00), and SYK (Ω = 1.00), consistent with their shared scaffold and directly corroborating the molecular docking results against EGFR, Bcl-2, and FabI. Kaempferol additionally predicted CYP19A1 (Ω = 1.00) and ESR1 (Ω = 1.00), suggesting hormonal pathway modulation. Rosmarinic acid predicted FYN kinase (Ω = 0.865), AKR1B1 (Ω = 0.865), and MMP1 (Ω = 0.865). Ursolic acid predicted RORC (Ω = 1.00), PTPN1 (Ω = 1.00), HSD11B1 (Ω = 1.00), and CDC25B (Ω = 1.00), consistent with its established triterpenoid target profile. Tanimoto similarity to approved drugs: kaempferol–acacetin (Tc = 0.78); ursolic acid–betulinic acid (Tc = 0.71).

##### 3.5.4.4 PASS biological activity prediction

PASS predicted broad pharmacological spectra for amentoflavone (95 activities, Pa >0.70) and kaempferol (100 activities), substantially richer than rosmarinic acid (27) and ursolic acid (33), consistent with the structural complexity of the flavonoid scaffold (Supplementary Table S6; Figure 12). Across both flavonoid leads, the highest-confidence shared predictions (Pa >0.90) included membrane integrity agonist (Pa = 0.969–0.974), *HIF1A* expression inhibitor (Pa = 0.969–0.973), kinase inhibitor (Pa = 0.959–0.964), *HMOX1* expression enhancer (Pa = 0.945–0.953), antimutagenic (Pa = 0.948–0.952), and peroxidase inhibitor (Pa = 0.948–0.956). Membrane integrity agonist and membrane permeability inhibitor predictions (Pa >0.96) are mechanistically consistent with the observed Gram-positive antibacterial selectivity. HMOX1 enhancement and NADPH oxidase inhibition (Pa = 0.889–0.918) provide predicted enzymatic mechanistic hypotheses consistent with the *in vitro* radical-scavenging reactivity observed. Apoptosis agonist (Pa = 0.881–0.903), *TP53* enhancement (Pa = 0.931–0.939), and antineoplastic (Pa = 0.791–0.826) predictions corroborate the antiproliferative results and docking predictions against EGFR, Bcl-2, and caspase-3. Rosmarinic acid showed a focused profile centred on membrane integrity (Pa = 0.956), antihypoxic (Pa = 0.921), JAK2 inhibition (Pa = 0.745), and antimutagenic activity (Pa = 0.722). Ursolic acid showed a distinct triterpenoid profile dominated by hepatoprotection (Pa = 0.961), NF-κB stimulation (Pa = 0.927), caspase-3 stimulation (Pa = 0.912), apoptosis agonism (Pa = 0.890), and antineoplastic activity (Pa = 0.857), providing spectra-based corroboration of its cytotoxic mechanism. Genotoxicity was flagged for amentoflavone (Pa = 0.852) and kaempferol (Pa = 0.843), consistent with known *in vitro* pro-oxidant activity of flavones at supratherapeutic concentrations, and not contradicting the favourable pkCSM AMES profiles; vascular toxicity was flagged for both (Pa = 0.773–0.788), warranting cardiovascular safety assessment in future preclinical studies.

**FIGURE 12:**
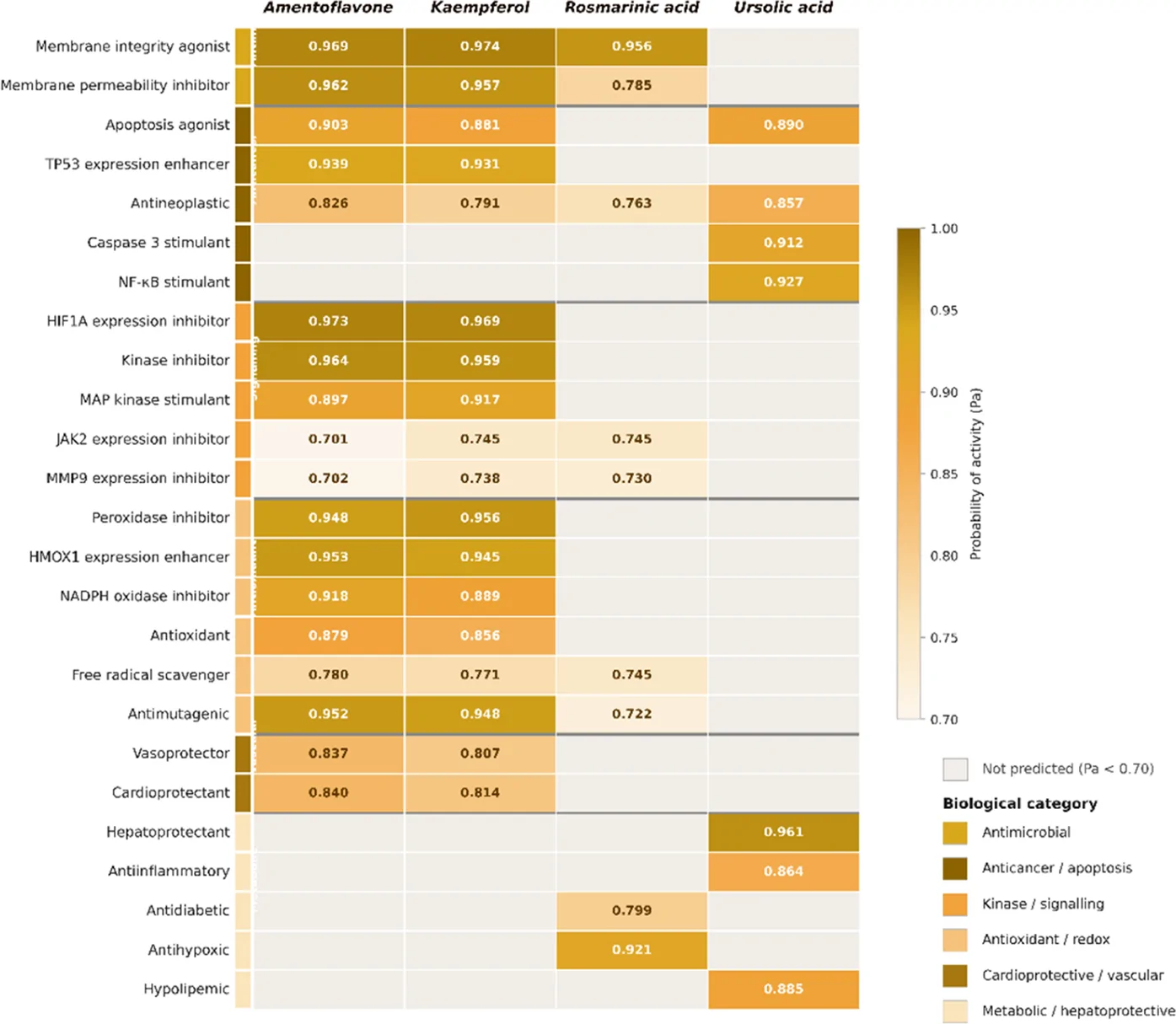
PASS-predicted biological activity heatmap for the four prioritized lead compounds from *A. decorticans* Boiss. Probability of activity (Pa) values generated by PASS Online (Pogodin et al., 2018) for amentoflavone, kaempferol, rosmarinic acid, and ursolic acid. Only activities with Pa >0.70 and Pa > Pi in at least one compound are shown. Colour intensity encodes Pa on a continuous amber scale from pale cream (Pa = 0.70) to deep gold (Pa = 1.00); grey cells indicate activities not predicted above threshold for that compound. Numerical Pa values are displayed in each cell. Activities are grouped by biological category (coloured side-bars, left). Genotoxicity and vascular toxicity (bottom rows) are adverse effect predictions; their Pa values reflect class-level pro-oxidant properties of flavones at supratherapeutic concentrations and do not contradict the favourable pkCSM mutagenicity profiles. Predictions performed for *H. sapiens* using isomeric SMILES strings (Supplementary Table S1) as input.

### 3.6 Principal component analysis

The principal component analyses (PCA) clearly discriminated the flowers, leaves, and stems of *A. decorticans* and revealed distinct relationships between their phytochemical composition and biological activities.

For the antioxidant PCA, PC1 and PC2 explained 58.8% and 41.2% of the total variance, respectively, and clearly separated the three plant organs. Because DPPH and ABTS were included as IC_50_ values, extracts positioned opposite to these vectors correspond to stronger radical-scavenging activity. The leaf extract was located in the opposite direction to both DPPH and ABTS, reflecting its lower IC_50_ values and superior scavenging capacity. Flowers occupied an intermediate position, whereas stems showed the closest overall association with the DPPH and ABTS directions, consistent with weaker activity and higher IC_50_ values. TAC followed a distinct direction and was most closely associated with leaves, followed by flowers, while stems were positioned on the opposite side of the plot, supporting the TAC ranking of leaves > flowers > stems. The flower cluster was mainly associated with caffeic, rosmarinic, trans-ferulic, and syringic acids and quercetin, whereas leaves were linked to several flavonoids and their glycosides, including rutin, epicatechin, and myricetin (Figure 13A).

**FIGURE 13:**
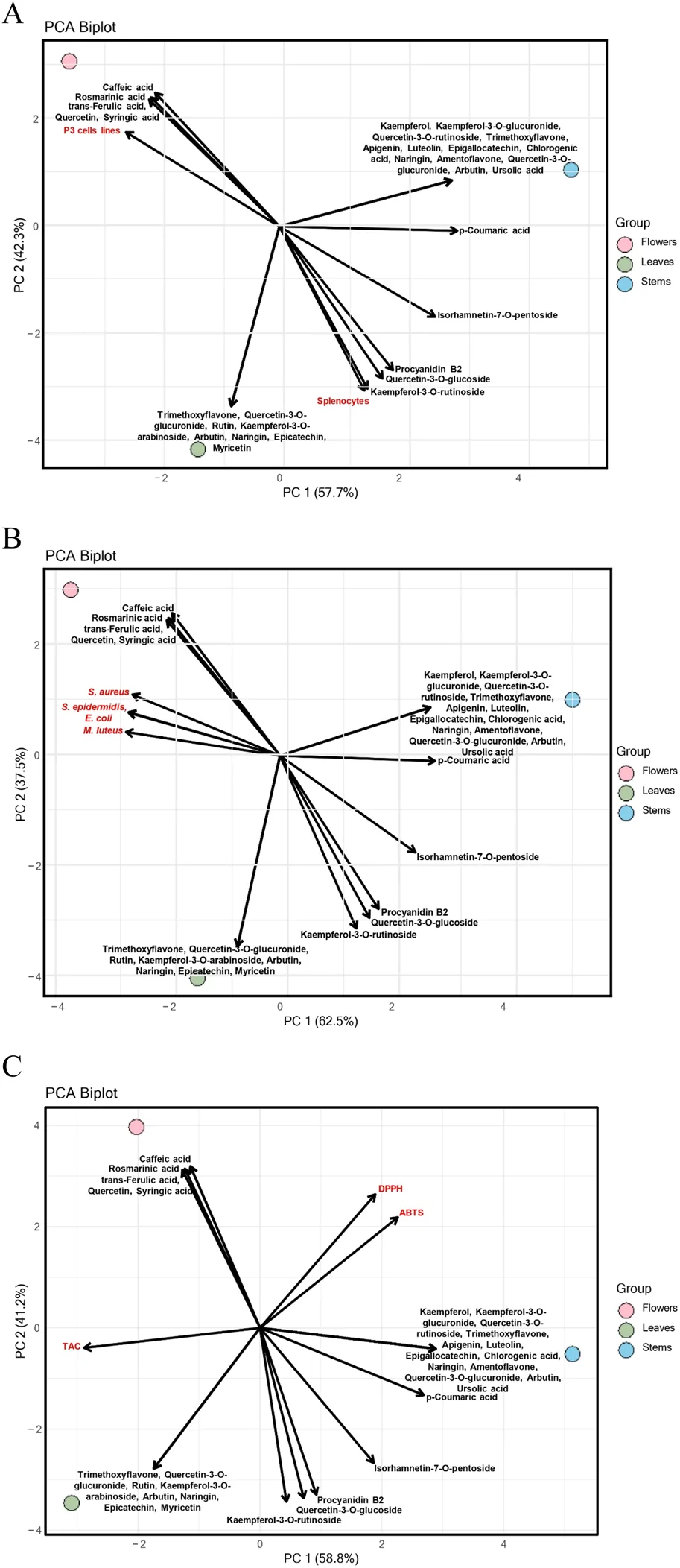
**(A)** Principal component analysis of chemical and antioxidant activity of *A. decorticans* hydroethanolic extracts. **(B)** Principal component analysis of chemical and antibacterial activity of *A. decorticans* hydroethanolic extracts. **(C)** Principal component analysis of chemical and cytotoxicity activity of *A. decorticans* hydroethanolic extracts.

In the antibacterial PCA, PC1 and PC2 accounted for 62.5% and 37.5% of the variance, respectively. The flower extract was positioned in the same direction as the DIZ vectors for *S. aureus*, *S. epidermidis*, *E. coli*, and *M. luteus*, indicating that the larger inhibition zones were primarily associated with the flower chemical profile. This association was particularly clear for *S. aureus*. Leaves occupied a secondary position and showed only a limited association with the DIZ variables, whereas stems were located on the opposite side of PC1, reflecting their weaker antibacterial performance. The loading pattern further linked the antibacterial activity of flowers to phenolic acids and quercetin, while stems were associated with p-coumaric acid and a broader group of flavonoids, flavonoid glycosides, and ursolic acid that showed a weaker relationship with DIZ-based activity (Figure 13B).

For the cytotoxicity PCA, PC1 and PC2 explained 57.7% and 42.3% of the variance, respectively, and again produced a complete separation of the three organs. As cytotoxicity was expressed as IC_50_, samples located opposite to an IC_50_ vector correspond to greater cytotoxic potency. The P3 cell-line IC_50_ vector pointed toward the flower cluster, indicating higher IC_50_ values and therefore lower cytotoxic potency for flowers. Stems were positioned in the opposite direction, consistent with lower P3 IC_50_ values and the greatest potency, while leaves showed an intermediate response. A different pattern was observed for splenocytes. The leaf extract was positioned closest to the splenocytes IC_50_ direction, suggesting the highest IC_50_ and weakest cytotoxicity, whereas flowers lay in the opposite direction and therefore showed the greatest cytotoxic potency, with stems occupying an intermediate position. P3 IC_50_ values were associated with the phenolic-acid-rich flower profile, whereas the splenocyte response was more closely related to procyanidin B2, quercetin-3-O-glucoside, and kaempferol-3-O-rutinoside (Figure 13C).

Overall, the PCA confirms a strong organ-driven segregation of *A. decorticans* extracts. Flowers were primarily associated with higher DIZ-based antibacterial activity and greater cytotoxicity toward splenocytes, leaves with superior *in vitro* radical-scavenging performance and the lowest splenocytes cytotoxicity, and stems with the highest cytotoxic potency against P3 cells but comparatively weaker antioxidant and antibacterial activities.

## 4 Discussion

Medicinal plant species synthesize diverse secondary metabolites that contribute to antioxidant, antimicrobial, anti-inflammatory, hypoglycemic, and cytotoxic effects and constitute important pharmacophoric scaffolds in modern drug discovery (Kumari et al., 2017; Negi et al., 2010). Within this framework, the present study provides an integrated phytochemical and biological characterization of hydroethanolic extracts from the flowers, leaves, and stems of *A. decorticans*, allowing both intra-organ comparison and interspecific contextualization within the genus *Adenocarpus*.

Extraction yields followed the order leaves > flowers > stems, consistent with the anatomical specialisation of these tissues: leaves accumulate substantial pools of soluble phenolics and their structure favours solvent diffusion, whereas lignified, cellulose-rich stems show reduced extractability (Wink, 1999; Alves-Ferreira et al., 2020). Comparative data across *Adenocarpus* species confirm that organ identity strongly influences extraction efficiency (Elgamouz and Bouzekri, 2021; Mechqoq et al., 2022; Benrazzouk et al., 2023).

Quantitative phenolic indices demonstrated pronounced organ specialisation, with TPC and TFC highest in leaves and TPrC enriched in stems, reflecting functional compartmentalisation between soluble flavonoid glycosides and condensed tannins. Comparison within the genus indicates that *A. decorticans* ranks among the more phenolic-rich species characterised to date, although such comparisons require caution because reported values differ in the basis of expression and in the calibration standard employed. Expressed per gram of extract, the leaf TPC recorded here (526.90 mg GAE/g) exceeds the values reported for *A. bacquei* leaves and stems (241.16 and 324.63 mg GAE/g) (Elgamouz and Bouzekri, 2021), *A. anagyrifolius* (326.80 mg GAE/g) (Benrazzouk et al., 2023) and *A. artemisiifolius* (301.68 mg GAE/g) (Mechqoq et al., 2022), while the flower and stem extracts (314.69 and 258.77 mg GAE/g) fall within that range. The leaf TFC (369.23 mg QE/g) likewise exceeded the corresponding values for these species. Data for *A. complicatus* (13.7 mg GAE/g and 36.67 mg RE/g) (Berber et al., 2014) are expressed on a different basis and in rutin equivalents respectively, and are therefore not compared directly.

UHPLC-ESI-MS profiling confirmed these quantitative trends and revealed clear metabolic compartmentalisation. Flowers were dominated by rosmarinic acid, indicating a specialised phenolic-acid centred profile (Sik et al., 2019; Essokne et al., 2012). Leaves exhibited the richest and most structurally diverse metabolome, characterised by abundant glycosylated flavonols and flavanones (rutin, myricetin, quercetin, kaempferol and naringin derivatives). Stems displayed the broadest structural heterogeneity, with elevated proanthocyanidins and flavone derivatives, aligning with their higher TPrC values and in agreement with the data by Essokne et al., for *A. telonensis* an *A. foliolosus* (Essokne et al., 2012).

Each organ accumulated the metabolite classes matched to its functional demands. Leaves, most exposed to ultraviolet radiation and photo-oxidative stress, were richest in flavonol and flavanone glycosides, which act as ultraviolet screens and radical scavengers, consistent with their highest total phenolic content and strongest radical-scavenging reactivity. Stems, requiring durable structural defence, were enriched in procyanidin and flavones, whose protein-precipitating capacity deters herbivores and pathogens and is compatible with both the highest proanthocyanidin content and the greatest antiproliferative potency of the stem extract. Flowers, short-lived reproductive organs facing a predominantly microbial challenge, were dominated by rosmarinic acid, whose membrane-disruptive action accords with their superior antibacterial activity and low selectivity between malignant and normal cells. This compartmentalisation, rather than a gradient of phenolic abundance, accounts for the divergence between total phenolic content and biological activity across organs. Leaves thus conform to the typical Adenocarpus flavonol-glycoside signature and stems to the genus-wide enrichment in proanthocyanidins, while the rosmarinic-acid centred profile of *A. decorticans* flowers has not previously been reported and refines the chemotaxonomic understanding of the genus.

Synthesizing these findings, leaves conform to the typical *Adenocarpus* flavonol-glycoside signature, stems reflect the genus-wide enrichment in proanthocyanidins, and *A. decorticans* flowers exhibit a distinctive rosmarinic-acid centered profile not previously reported, refining the chemotaxonomic understanding of the genus.

The biological activities reported below were determined on crude, unfractionated extracts, so activity is a property of the mixture and the correspondences drawn between composition and activity indicate which constituents merit investigation rather than establishing that they are responsible for the effects observed. Extract activity may arise from additive, synergistic or antagonistic interactions among constituents, and the %AUC values obtained by targeted UHPLC-ESI-MS are semi-quantitative descriptors of relative contribution within each profile that do not establish that the most abundant constituents are the active ones. Minor constituents, including compound classes outside the detection scope of the targeted method, may contribute disproportionately. Resolution of these questions requires bioassay-guided fractionation and testing of isolated compounds alone and in combination, which lies beyond the scope of the present study.

Radical-scavenging and reducing reactivity was assessed using complementary assays (DPPH, ABTS and TAC) encompassing hydrogen atom transfer and single-electron transfer mechanisms. All extracts showed IC_50_ values below 50 μg/mL in DPPH, with a consistent hierarchy of leaves > flowers > stems across assays that mirrors phenolic distribution. Leaf extracts, enriched in flavonol and flavanone glycosides, demonstrated the highest reactivity, consistent with the electron-donating properties associated with hydroxyl substitution patterns and conjugated aromatic systems. The intermediate activity of floral extracts is compatible with the abundance of rosmarinic acid in this extract, a phenolic ester reported to stabilise free radicals via resonance delocalisation, while the proanthocyanidin-rich stem extract retained appreciable reactivity reflecting the cumulative electron-donating capacity of condensed tannins.

Compared with other *Adenocarpus* species, *A. decorticans* showed competitive or superior reactivity. Its DPPH IC_50_ values were lower than those reported for *A. anagyrifolius* (90.43 μg/mL) (Benrazzouk et al., 2023), *A. artemisiifolius* (58.81 μg/mL) and *A. complicatus* (474.95 μg/mL) (Berber et al., 2014), and comparable to *A. bacquei* (leaf 24.07 μg/mL, stem 20.32 μg/mL) (Elgamouz and Bouzekri, 2021), while *A. mannii* displayed exceptionally strong activity approaching that of ascorbic acid (2.82 μg/mL) (Ndjateu et al., 2014). In the ABTS assay the values recorded here (4.41–9.90 μg/mL across organs) were markedly below those reported for *A. artemisiifolius* (97.1 μg/mL) *A. mannii* (361.30 μg/mL) and *A. complicatus* (474.95 μg/mL) (Mechqoq et al., 2022).

Hydroethanolic extracts showed moderate, bactericidal and Gram-positive-selective antibacterial activity, with flowers most active, followed by leaves and stems. The higher sensitivity of Gram-positive strains is attributable to their thick peptidoglycan layer and absence of an outer membrane, whereas the lipopolysaccharide-rich outer membrane of Gram-negative bacteria restricts penetration of many antimicrobial agents (Zhao et al., 2001), as illustrated by the resistance of *P. aeruginosa* to all extracts despite its susceptibility to chlortetracycline.

Antibacterial potency did not parallel total phenolic or flavonoid content: although leaves contained the highest TPC and TFC, floral extracts were more active, indicating that efficacy depends on specific constituents rather than global phenolic abundance. The flower extract was dominated by rosmarinic acid at 80.60% of total AUC, and this phenolic ester provides a plausible explanation for the observed activity, since it acts through disruption of membrane integrity and antibiofilm effects and displays well documented anti-inflammatory activity across *in vitro* and *in vivo* models (Luo et al., 2020). This chemical dominance converges with the ethnomedicinal record obtained in our survey of the Oriental region, in which flowers of *A. decorticans* were the material reported by informants, and with the present data, in which the flower extract was the most antibacterially active organ and exerted bactericidal activity against Gram-positive strains at MIC values of 0.78–2.08 mg/mL. We interpret this convergence as mechanistic plausibility for the documented traditional use rather than as evidence of that use, which rests on the survey data alone. Stem extracts, enriched in polymeric proanthocyanidins, showed weaker activity, consistent with the limited diffusion and reduced target accessibility of high-molecular-weight tannins.

Comparative data within the genus remain limited. Ndjateu et al. (2014) reported moderate activity for ethanol leaf extracts of *A. mannii*, with MIC values around 0.16 mg/mL against *S. aureus*, *E. coli* and *E. faecalis*, while studies on endophytic fungi associated with *A. foliolosus* describe metabolites of fungal rather than plant origin (Dai et al., 2005). Following bioassay-guided fractionation, *A. decorticans* flower extracts represent a promising starting point for the development of Gram-positive-selective antibacterial leads.

Cytotoxic evaluation against P3 myeloma cells and primary splenocytes revealed marked organ-dependent variation, and the two cell types gave inverted organ rankings. Stem extracts exhibited the strongest antiproliferative effect on P3 cells, followed by leaf extracts, whereas flower extracts were least active. Against normal splenocytes the order was reversed, with flower extracts the most cytotoxic and leaf extracts the least. Stem extracts consequently combined the highest antiproliferative potency with the highest selectivity index (SI = 1.97), while the flower extract returned an SI of 0.16 and is therefore not a viable antiproliferative candidate. This is mechanistically coherent with its composition, since the dominance of rosmarinic acid is compatible with a membrane-disruptive mode of action, which would be expected to show little selectivity between cell types and would be consistent with both the superior antibacterial activity of this organ and its indiscriminate toxicity toward membrane-fragile primary lymphocytes.

These patterns align with the metabolite profiles. Stem extracts contained structurally diverse constituents including flavones, biflavonoids, triterpenoids and elevated procyanidin levels, many associated with antiproliferative mechanisms such as mitochondrial dysfunction, cell cycle arrest and apoptosis induction, whereas leaf extracts dominated by glycosylated flavonoids showed intermediate potency, possibly reflecting lower cellular uptake.

Radical scavenging was observed at 4–22 μg/mL whereas the reduction in cell viability required 343–909 μg/mL, so the two activities operate in distinct concentration regimes and are unlikely to share a common mechanism. Polyphenols are moreover reported to act as pro-oxidants rather than radical scavengers at high concentrations. Whether redox perturbation contributes to the antiproliferative effect was not investigated here and would require direct measurement of intracellular reactive oxygen species. Cytotoxicity in *A. decorticans* is therefore not predicted by total phenolic concentration but appears to depend on the identity of the constituents present, and although stem extracts offer the most favourable combination of potency and selectivity, the selectivity achieved remains below the conventional threshold, so bioassay-guided fractionation will be required before any antiproliferative candidate can be advanced.

Molecular docking was used to compare the binding potential of the identified metabolites against targets relevant to the measured activities, with the highest-ranking complexes carried forward to molecular dynamics simulation. These approaches are predictive rather than confirmatory: docking scores estimate steric and electrostatic complementarity under simplified assumptions and do not demonstrate target engagement, which requires enzymatic or cell-based validation. The computational results are accordingly interpreted as generating hypotheses about the molecular basis of the measured activities rather than as evidence of them.

Within these limits, the compounds ranking highest across all target classes were flavonoid glycosides and proanthocyanidins rather than simple phenolic acids, paralleling the experimental observation that potency did not track total phenolic content. Procyanidin B2, rutin and naringin bound favourably to both antibacterial and anticancer targets, and amentoflavone displayed the broadest multi-target profile. Two qualifications apply. Amentoflavone was detected only in the stem extract and accounted for 1.06% of total peak area, so its predicted binding cannot by itself account for the activity of that extract; predicted affinity indicates which scaffolds merit isolation but does not weight a compound’s contribution to a complex mixture. Conversely, rosmarinic acid, which constitutes 80.60% of the flower extract and provides the most plausible explanation for its antibacterial activity, was not among the ligands prioritised by docking, so the computational ranking and the experimental antibacterial result address different constituents and should not be read as mutually confirming.

Molecular dynamics simulations tested whether the docked poses persisted over time. The EGFR-amentoflavone complex proved the more stable, equilibrating after approximately 30 ns and maintaining moderate residue fluctuations, a constant radius of gyration and one to three persistent hydrogen bonds. The Bcl-2-amentoflavone complex showed greater backbone drift, higher residue flexibility and only intermittent hydrogen bonding, indicating a less well accommodated pose. In both systems the ligand remained associated with the binding site (ligand RMSD 0.13–0.18 nm). These results identify the biflavonoid scaffold, and EGFR in particular, as the most defensible starting point for isolation and direct target validation, while neither EGFR nor Bcl-2 modulation was tested experimentally here.

Target prediction and biological activity spectra provided orthogonal support for the same scaffold. Amentoflavone and kaempferol shared 26 predicted human targets including NOX4, AKR1B1, CDK5, FLT3 and SYK, while ursolic acid returned a distinct triterpenoid target set. PASS predictions for the flavonoid leads centred on membrane integrity and permeability, mechanistically consistent with the Gram-positive selectivity observed, and on apoptosis agonism and TP53 enhancement, paralleling the antiproliferative results. These convergences are informative because the three approaches rest on different premises, so agreement between them is not circular; they nonetheless remain predictions, and the genotoxicity and vascular toxicity flags for both flavonoid leads warrant experimental verification.

ADMET profiling produced the most consequential caveat of the computational analysis. Fifteen compounds complied fully with Lipinski’s rule of five, but the fourteen glycosylated constituents systematically exceeded molecular weight and hydrogen-bonding thresholds and showed low predicted intestinal absorption as intact molecules, consistent with their established biotransformation to aglycones by gut microbiota. Several compounds that dominate these extracts and rank highest in docking are therefore unlikely to reach systemic circulation unchanged. The activities measured here *in vitro* should not be extrapolated to oral efficacy without pharmacokinetic study, and the aglycone forms are the more realistic starting points for development.

Principal component analysis of the combined chemical and biological data recovered the same organ-driven structure obtained by univariate comparison, with flowers aligning with antibacterial performance, leaves with radical-scavenging reactivity and stems with cytotoxic potency. Because the analysis comprises three samples, two components necessarily account for the entire variance, so the ordination is a descriptive visualisation of the organ-level structure rather than a statistical test of it.

## 5 Conclusion

This study provides the first organ-resolved phytochemical, biological and computational characterisation of *A. decorticans*. UHPLC-ESI-MS profiling revealed pronounced metabolic compartmentalisation, with flavonol- and flavanone-rich leaves, rosmarinic acid-dominated flowers and proanthocyanidin-enriched stems, and the biological activities followed this distribution rather than total phenolic content. Leaf extracts showed the strongest *in vitro* radical-scavenging reactivity, flower extracts the highest antibacterial activity, bactericidal and selective for Gram-positive strains, and stem extracts the most potent antiproliferative activity together with the highest selectivity index; selectivity nonetheless remained modest across all organs, and the flower extract was preferentially toxic to normal splenocytes. Molecular docking and 100 ns molecular dynamics simulations generated mechanistic hypotheses for these findings, ranking flavonoid glycosides and proanthocyanidins, particularly amentoflavone, rutin, naringin and procyanidin B2, as high-affinity binders of antibacterial and anticancer targets, with the EGFR-amentoflavone complex the most dynamically stable. ADMET profiling indicated favourable drug-likeness for the aglycone leads while predicting poor intestinal absorption for the glycosylated constituents that dominate the extracts. Because all assays were performed on crude extracts, these correspondences identify candidate constituents rather than establishing their contribution. Bioassay-guided fractionation, isolation of the active constituents, and mechanistic and *in vivo* validation are the necessary next steps toward therapeutic development.

## Author’s note

Material was collected at a single site and phenological stage and extracted with one hydroethanolic solvent, leaving seasonal and geographical variation and less polar constituents uncaptured.

The UHPLC-ESI-MS profile derives from a single injection per organ, so %AUC values are semi-quantitative descriptors of relative contribution within each profile, and without co-injected authentic standards all constituents are Level 3 annotations.

The targeted single-ion-monitoring method excluded alkaloids, reported in this species and co-extracted by hydroethanolic maceration, so their contribution cannot be ruled out.

Folin-Ciocalteu measures total reducing capacity rather than phenolics specifically, and all quantitative values are expressed per gram of extract.

DPPH, ABTS and phosphomolybdenum are chemical redox tests that do not establish antioxidant activity in the pharmacological sense, which requires the cell-based or *in vivo* models not performed here.

Antibacterial activity was determined on crude extracts against ATCC reference strains rather than clinical isolates, the MIC values are moderate and underestimate the potency of individual constituents, and the diffusion data, carrying 10 mg extract per disc against 30 µg antibiotic, served only as a preliminary screen.

MTT cannot separate cytotoxic from cytostatic effects except in the non-proliferating splenocyte system, testing was confined to one murine myeloma line with primary splenocytes as the normal-cell comparator under a different seeding protocol, and all selectivity indices fell below 2.

Because all assays used crude extracts, composition-activity correspondences identify candidates rather than establish contribution; the computational analyses are predictive only, with amentoflavone a minor constituent and rosmarinic acid absent from the docking set, so bioassay-guided fractionation and *in vivo* validation remain necessary.

## Data Availability

All data generated or analysed during this study are included in this article and its Supplementary Material. Further enquiries can be directed to the corresponding author.

## Supplementary material

Supplementary Table S1 Isomeric SMILES strings of the 29 phytochemicals identified by UHPLC-ESI-MS/SIM, used as input for ADMET, target and PASS predictions.

Supplementary Table S2 SwissADME physicochemical and pharmacokinetic descriptors of the 29 identified phytochemicals.

Supplementary Table S3 pkCSM-predicted toxicity endpoints of the 29 identified phytochemicals.

Supplementary Table S4 Multi-filter drug-likeness compliance and composite bioavailability score (F20%) of the 29 identified phytochemicals.

Supplementary Table S5 SwissTargetPrediction human protein targets of the four prioritized lead compounds.

Supplementary Table S6 PASS Online predicted biological activity spectra of the four prioritized lead compounds. No line numbers or tracked changes are visible in the files, and no material has been previously deposited elsewhere.
