# Supplementary material for: Organ-dependent phytochemical and pharmacological specialization in the endemic Adenocarpus decorticans Boiss
Source: Front Pharmacol. 2026 Oct 8;17:1919944. doi: 10.3389/fphar.2026.1919944 (PMC13711306; doi:10.3389/fphar.2026.1919944)
Supplement: Supplementary file 1 [file Table1.docx]

**Table S1.** Isomeric SMILES strings for the 29 phytochemicals identified in *A. decorticans* Boiss. by UHPLC-ESI-MS, used as input for SwissADME and PASS Online analyses.

| **No.** | **Compound** | **Organ** | **Isomeric SMILES** |
| --- | --- | --- | --- |
| 1 | *Rosmarinic acid* | F | OC(=O)[C@@H](Cc1ccc(O)c(O)c1)OC(=O)/C=C/c1ccc(O)c(O)c1 |
| 2 | *trans-Ferulic acid* | F,L,S | COc1cc(/C=C/C(=O)O)ccc1O |
| 3 | *Ferulic acid* | F | COc1cc(CC(=O)O)ccc1O |
| 4 | *Syringic acid* | F | COc1cc(C(=O)O)cc(OC)c1O |
| 5 | *Quercetin* | F | O=c1c(O)c(-c2ccc(O)c(O)c2)oc2cc(O)cc(O)c12 |
| 6 | *Caffeic acid* | F,L,S | OC(=O)/C=C/c1ccc(O)c(O)c1 |
| 7 | *Myricetin* | L | O=c1c(O)c(-c2cc(O)c(O)c(O)c2)oc2cc(O)cc(O)c12 |
| 8 | *Procyanidin B2* | L,S | Oc1ccc([C@@H]2Oc3cc(O)cc(O)c3C[C@H]2O)cc1O |
| 9 | *Kaempferol-3-O-glucoside* | L,S | O=c1c(O[C@@H]2O[C@@H](CO)[C@@H](O)[C@H](O)[C@H]2O)c(-c2ccc(O)cc2)oc2cc(O)cc(O)c12 |
| 10 | *Naringin* | L,S | O=C1CC(c2ccc(OC3O[C@@H](CO)[C@H](O)[C@H](O)[C@@H]3O)cc2)Oc2cc(O[C@@H]3O[C@H](CO)[C@H](O)[C@H](O)[C@@H]3O)cc(O)c21 |
| 11 | *Rutin* | L | O=c1c(O[C@@H]2O[C@@H](CO[C@@H]3O[C@H](C)[C@@H](O)[C@H](O)[C@H]3O)[C@@H](O)[C@H](O)[C@H]2O)c(-c2ccc(O)c(O)c2)oc2cc(O)cc(O)c12 |
| 12 | *Isorhamnetin-7-O-pentose* | L,S | COc1c(-c2oc3cc(O[C@@H]4OCC(O)[C@H](O)[C@@H]4O)cc(O)c3c(=O)c2O)ccc(O)c1O |
| 13 | *Quercetin-3-O-hexose-deoxyhex* | L,S | O=c1c(O[C@@H]2O[C@H](C)[C@@H](O)[C@H](O)[C@H]2O)c(-c2ccc(O)c(O)c2)oc2cc(O)cc(O)c12 |
| 14 | *Quercetin-3-O-glucoside* | L,S | O=c1c(O[C@@H]2O[C@@H](CO)[C@@H](O)[C@H](O)[C@H]2O)c(-c2ccc(O)c(O)c2)oc2cc(O)cc(O)c12 |
| 15 | *Kaempferol-3-O-hexose-deoxyhex* | L,S | O=c1c(O[C@@H]2O[C@H](C)[C@@H](O)[C@H](O)[C@H]2O)c(-c2ccc(O)cc2)oc2cc(O)cc(O)c12 |
| 16 | *Arbutin* | L,S | OC[C@H]1O[C@@H](Oc2ccc(O)cc2)[C@H](O)[C@@H](O)[C@@H]1O |
| 17 | *Quercetin-3-O-glucuronic acid* | L,S | O=c1c(O[C@@H]2O[C@@H](C(=O)O)[C@@H](O)[C@H](O)[C@@H]2O)c(-c2ccc(O)c(O)c2)oc2cc(O)cc(O)c12 |
| 18 | *Trimethoxyflavone* | L,S | COc1ccc(-c2cc(=O)c3c(OC)cc(OC)cc3o2)cc1 |
| 19 | *Epicatechin* | L | Oc1ccc([C@@H]2Oc3cc(O)cc(O)c3C[C@@H]2O)cc1O |
| 20 | *p-Coumaric acid* | L,S | OC(=O)/C=C/c1ccc(O)cc1 |
| 21 | *Kaempferol-3-O-pentose* | L | O=c1c(O[C@@H]2O[C@H](CO)[C@@H](O)[C@@H](O)[C@@H]2O)c(-c2ccc(O)cc2)oc2cc(O)cc(O)c12 |
| 22 | *Kaempferol* | S | O=c1c(O)c(-c2ccc(O)cc2)oc2cc(O)cc(O)c12 |
| 23 | *Luteolin* | S | O=c1cc(-c2ccc(O)c(O)c2)oc2cc(O)cc(O)c12 |
| 24 | *Apigenin* | S | O=c1cc(-c2ccc(O)cc2)oc2cc(O)cc(O)c12 |
| 25 | *Ursolic acid* | S | C[C@@H]1CC[C@@]2(CC[C@@]3(C(=CC[C@H]4[C@]3(CC[C@@H]5[C@@]4(CC[C@@H](C5(C)C)O)C)C)[C@@H]2[C@H]1C)C)C(=O)O |
| 26 | *Amentoflavone* | S | O=c1cc(-c2ccc(-c3oc4cc(O)cc(O)c4c(=O)c3O)cc2)oc2cc(O)cc(O)c12 |
| 27 | *Chlorogenic acid* | S | O=C(/C=C/c1ccc(O)c(O)c1)O[C@@H]1CC(O)(C(=O)O)[C@H](O)[C@@H](O)[C@@H]1O |
| 28 | *Epigallocatechin* | S | Oc1cc(O)cc2c1O[C@H](c1cc(O)c(O)c(O)c1)CC2O |
| 29 | *Kaempferol-3-O-glucuronic acid* | S | O=c1c(O[C@@H]2O[C@@H](C(=O)O)[C@@H](O)[C@H](O)[C@@H]2O)c(-c2ccc(O)cc2)oc2cc(O)cc(O)c12 |

**Table S2.** SwissADME-predicted ADMET physicochemical and pharmacokinetic properties of the 29 phytochemicals identified in *A.decorticans*.

| **Compound** | **Organ** | **MW (g/mol)** | **LogP** | **GIA** | **BBB** | **Ro5** | **F20%** | **Brenk** | **CYP3A4 inhib.** |
| --- | --- | --- | --- | --- | --- | --- | --- | --- | --- |
| *Rosmarinic acid* | F | 360.3 | 1.58 | Low | No | Pass | 0.60 | 2 | No |
| *trans-Ferulic acid* | F,L,S | 194.2 | 1.36 | High | Yes | Pass | 0.80 | 1 | No |
| *Ferulic acid* | F | 182.2 | 0.88 | High | No | Pass | 0.80 | 0 | No |
| *Syringic acid* | F | 198.2 | 0.99 | High | No | Pass | 0.80 | 0 | No |
| *Quercetin* | F | 302.2 | 1.23 | High | No | Pass | 1.00 | 1 | Yes |
| *Caffeic acid* | F,L,S | 180.2 | 0.93 | High | No | Pass | 0.80 | 2 | No |
| *Myricetin* | L | 318.2 | 0.79 | Low | No | Fail(1) | 0.20 | 1 | Yes |
| *Procyanidin B2* | L,S | 290.3 | 0.83 | High | No | Pass | 1.00 | 1 | No |
| *Kaempferol-3-O-glucoside* | L,S | 448.4 | -0.02 | Low | No | Fail(2) | 0.20 | 0 | No |
| *Naringin* | L,S | 596.5 | -1.42 | Low | No | Fail(3) | 0.00 | 0 | No |
| *Rutin* | L | 610.5 | -1.42 | Low | No | Fail(3) | 0.00 | 1 | No |
| *Isorhamnetin-7-O-pentose* | L,S | 464.4 | -0.04 | Low | No | Fail(2) | 0.20 | 1 | No |
| *Quercetin-3-O-hexose-deoxyhex* | L,S | 448.4 | 0.26 | Low | No | Fail(2) | 0.20 | 1 | No |
| *Quercetin-3-O-glucoside* | L,S | 464.4 | -0.49 | Low | No | Fail(2) | 0.00 | 1 | No |
| *Kaempferol-3-O-hexose-deoxyhex* | L,S | 432.4 | 0.70 | Low | No | Fail(1) | 0.20 | 0 | No |
| *Arbutin* | L,S | 272.2 | -0.77 | High | No | Pass | 0.80 | 0 | No |
| *Quercetin-3-O-glucuronic acid* | L,S | 478.4 | -0.51 | Low | No | Fail(2) | 0.00 | 1 | No |
| *Trimethoxyflavone* | L,S | 312.3 | 3.10 | High | Yes | Pass | 1.00 | 0 | Yes |
| *Epicatechin* | L | 290.3 | 0.83 | High | No | Pass | 1.00 | 1 | No |
| *p-Coumaric acid* | L,S | 164.2 | 1.26 | High | Yes | Pass | 0.80 | 1 | No |
| *Kaempferol-3-O-pentose* | L | 448.4 | 0.00 | Low | No | Fail(2) | 0.20 | 0 | No |
| *Kaempferol* | S | 286.2 | 1.58 | High | No | Pass | 1.00 | 0 | Yes |
| *Luteolin* | S | 286.2 | 1.73 | High | No | Pass | 1.00 | 1 | Yes |
| *Apigenin* | S | 270.2 | 2.11 | High | No | Pass | 1.00 | 0 | Yes |
| *Ursolic acid* | S | 456.7 | 5.93 | Low | No | Fail(1) | 0.20 | 1 | No |
| *Amentoflavone* | S | 446.4 | 2.76 | Low | No | Pass | 0.40 | 0 | No |
| *Chlorogenic acid* | S | 370.3 | -1.08 | Low | No | Fail(1) | 0.00 | 2 | No |
| *Epigallocatechin* | S | 306.3 | 0.64 | High | No | Fail(1) | 0.60 | 1 | No |
| *Kaempferol-3-O-glucuronic acid* | S | 462.4 | -0.08 | Low | No | Fail(2) | 0.20 | 0 | No |

**Table S3**. pkCSM-predicted toxicity profiles for the 29 phytochemicals identified in *A.decorticans***.**

| **Compound** | **Organ** | **AMES** | **Hepatotox.** | **hERG I** | **hERG II** | **LD₅₀ (log mol/kg)** | **Skin sensit.** |
| --- | --- | --- | --- | --- | --- | --- | --- |
| *Amentoflavone* | S | No | No | No | **Yes** | 2.82 | No |
| *Apigenin* | S | No | No | No | **Yes** | 2.43 | No |
| *Arbutin* | L,S | No | No | No | No | 1.91 | No |
| *Caffeic acid* | F,L,S | **Yes** | No | No | No | 2.02 | No |
| *Chlorogenic acid* | S | No | No | No | No | 2.39 | No |
| *Epicatechin* | L | **Yes** | No | No | No | 2.16 | No |
| *Epigallocatechin* | S | No | No | No | No | 2.30 | No |
| *Ferulic acid* | F | No | No | No | No | 2.17 | No |
| *Isorhamnetin-7-O-pentose* | L,S | **Yes** | No | No | **Yes** | 2.78 | No |
| *Kaempferol* | S | **Yes** | No | No | No | 2.35 | No |
| *Kaempferol-3-O-glucoside* | L,S | **Yes** | No | No | **Yes** | 3.06 | No |
| *Kaempferol-3-O-hexose-deoxyhex* | L,S | **Yes** | No | No | **Yes** | 3.08 | No |
| *Kaempferol-3-O-pentose* | L | **Yes** | No | No | **Yes** | 3.06 | No |
| *Luteolin* | S | **Yes** | No | No | No | 2.71 | No |
| *Myricetin* | L | No | No | No | No | 2.50 | No |
| *Naringin* | L,S | **Yes** | No | No | No | 2.46 | No |
| *p-Coumaric acid* | L,S | No | No | No | No | 2.07 | No |
| *Procyanidin B2* | L,S | **Yes** | No | No | No | 2.16 | No |
| *Quercetin* | F | No | No | No | No | 2.44 | No |
| *Quercetin-3-O-glucoside* | L,S | **Yes** | No | No | **Yes** | 2.82 | No |
| *Quercetin-3-O-glucuronic acid* | L,S | **Yes** | No | No | No | 2.68 | No |
| *Quercetin-3-O-hexose-deoxyhex* | L,S | **Yes** | No | No | **Yes** | 2.97 | No |
| *Rosmarinic acid* | F | No | No | No | No | 2.24 | No |
| *Rutin* | L | **Yes** | No | No | **Yes** | 2.48 | No |
| *Syringic acid* | F | No | No | No | No | 1.97 | No |
| *trans-Ferulic acid* | F,L,S | No | No | No | No | 2.28 | No |
| *Trimethoxyflavone* | L,S | **Yes** | No | No | **Yes** | 2.41 | No |
| *Ursolic acid* | S | No | **Yes** | No | No | 2.35 | No |

**Table S4.** *Multi-filter drug-likeness compliance and composite bioavailability score (F20%) for the 29 phytochemicals identified in A.decorticans***.**

| **Compound** | **Organ** | **Ro5** | **Ghose** | **Veber** | **Egan** | **Muegge** | **F20%** | **Main violations** |
| --- | --- | --- | --- | --- | --- | --- | --- | --- |
| *Rosmarinic acid* | F | Pass | Fail | Fail | Fail | Pass | 0.60 | Low GIA |
| *trans-Ferulic acid* | F,L,S | Pass | Pass | Pass | Pass | Fail | 0.80 | Muegge MR |
| *Ferulic acid* | F | Pass | Pass | Pass | Pass | Fail | 0.80 | Muegge MR |
| *Syringic acid* | F | Pass | Pass | Pass | Pass | Fail | 0.80 | Muegge MR |
| *Quercetin* | F | Pass | Pass | Pass | Pass | Pass | 1.00 | — |
| *Caffeic acid* | F,L,S | Pass | Pass | Pass | Pass | Fail | 0.80 | Muegge MR |
| *Myricetin* | L | Fail | Pass | Fail | Fail | Fail | 0.20 | MW, TPSA |
| *Procyanidin B2* | L,S | Pass | Pass | Pass | Pass | Pass | 1.00 | — |
| *Kaempferol-3-O-glucoside* | L,S | Fail | Pass | Fail | Fail | Fail | 0.20 | MW, HBD |
| *Naringin* | L,S | Fail | Fail | Fail | Fail | Fail | 0.00 | MW, HBD, HBA |
| *Rutin* | L | Fail | Fail | Fail | Fail | Fail | 0.00 | MW, HBD, HBA |
| *Isorhamnetin-7-O-pentose* | L,S | Fail | Pass | Fail | Fail | Fail | 0.20 | MW, HBD |
| *Quercetin-3-O-hexose-deoxyhex* | L,S | Fail | Pass | Fail | Fail | Fail | 0.20 | MW |
| *Quercetin-3-O-glucoside* | L,S | Fail | Fail | Fail | Fail | Fail | 0.00 | MW, HBD |
| *Kaempferol-3-O-hexose-deoxyhex* | L,S | Fail | Pass | Fail | Fail | Fail | 0.20 | MW |
| *Arbutin* | L,S | Pass | Fail | Pass | Pass | Pass | 0.80 | Ghose MR |
| *Quercetin-3-O-glucuronic acid* | L,S | Fail | Fail | Fail | Fail | Fail | 0.00 | MW, HBD |
| *Trimethoxyflavone* | L,S | Pass | Pass | Pass | Pass | Pass | 1.00 | — |
| *Epicatechin* | L | Pass | Pass | Pass | Pass | Pass | 1.00 | — |
| *p-Coumaric acid* | L,S | Pass | Pass | Pass | Pass | Fail | 0.80 | Muegge MR |
| *Kaempferol-3-O-pentose* | L | Fail | Pass | Fail | Fail | Fail | 0.20 | MW, HBD |
| *Kaempferol* | S | Pass | Pass | Pass | Pass | Pass | 1.00 | — |
| *Luteolin* | S | Pass | Pass | Pass | Pass | Pass | 1.00 | — |
| *Apigenin* | S | Pass | Pass | Pass | Pass | Pass | 1.00 | — |
| *Ursolic acid* | S | Fail | Fail | Pass | Fail | Fail | 0.20 | LogP, MW |
| *Amentoflavone* | S | Pass | Pass | Fail | Fail | Fail | 0.40 | TPSA, HBD |
| *Chlorogenic acid* | S | Fail | Fail | Fail | Fail | Fail | 0.00 | MW, HBD |
| *Epigallocatechin* | S | Fail | Pass | Pass | Pass | Fail | 0.60 | Ro5 MW |
| *Kaempferol-3-O-glucuronic acid* | S | Fail | Pass | Fail | Fail | Fail | 0.20 | MW, HBD |

**Table S5.** SwissTargetPrediction-predicted human protein targets for the four prioritized lead compounds from *A. decorticans* (Homo sapiens, Ω ≥ 0.30 shown).

| **Compound** | **Target (gene name)** | **Target class** | **Ω** |
| --- | --- | --- | --- |
| *Amentoflavone* | NOX4 (NADPH oxidase 4) | Enzyme | **1.000** |
| Amentoflavone | AKR1B1 (Aldose reductase) | Enzyme | **1.000** |
| Amentoflavone | CDK5 (Cyclin-dependent kinase 5) | Kinase | **1.000** |
| Amentoflavone | FLT3 (Receptor tyrosine kinase) | Kinase | **1.000** |
| Amentoflavone | SYK (Spleen tyrosine kinase) | Kinase | **1.000** |
| Amentoflavone | ALOX5 (Arachidonate 5-lipoxygenase) | Oxidoreductase | **1.000** |
| Amentoflavone | TNKS / TNKS2 (Tankyrase 1/2) | Enzyme | **1.000** |
| Amentoflavone | CDK1/CCNB1 complex | Kinase | **1.000** |
| Amentoflavone | ADORA1 (Adenosine A1 receptor) | GPCR | **1.000** |
| Amentoflavone | FYN (Tyrosine-protein kinase Fyn) | Kinase | 0.865 |
| *Kaempferol* | NOX4 (NADPH oxidase 4) | Enzyme | **1.000** |
| Kaempferol | AKR1B1 (Aldose reductase) | Enzyme | **1.000** |
| Kaempferol | CDK5 (Cyclin-dependent kinase 5) | Kinase | **1.000** |
| Kaempferol | FLT3 (Receptor tyrosine kinase) | Kinase | **1.000** |
| Kaempferol | SYK (Spleen tyrosine kinase) | Kinase | **1.000** |
| Kaempferol | CYP19A1 (Aromatase) | Cytochrome P450 | **1.000** |
| Kaempferol | ESR1 (Estrogen receptor α) | Nuclear receptor | **1.000** |
| Kaempferol | ACHE (Acetylcholinesterase) | Hydrolase | **1.000** |
| Kaempferol | CDK1/CCNB1 complex | Kinase | **1.000** |
| Kaempferol | MAOA (Monoamine oxidase A) | Oxidoreductase | **1.000** |
| *Rosmarinic acid* | FYN (Tyrosine-protein kinase Fyn) | Kinase | 0.865 |
| Rosmarinic acid | AKR1B1 (Aldose reductase) | Enzyme | 0.865 |
| Rosmarinic acid | TTR (Transthyretin) | Secreted protein | 0.865 |
| Rosmarinic acid | MMP1 (Matrix metalloproteinase 1) | Protease | 0.865 |
| *Ursolic acid* | RORC (Nuclear receptor ROR-γ) | Nuclear receptor | **1.000** |
| Ursolic acid | POLB (DNA polymerase β) | Enzyme | **1.000** |
| Ursolic acid | PTPN1 (PTP1B) | Phosphatase | **1.000** |
| Ursolic acid | PTPN2 (TC-PTP) | Phosphatase | **1.000** |
| Ursolic acid | HSD11B1 (11β-HSD1) | Enzyme | **1.000** |
| Ursolic acid | CDC25B (Cell division phosphatase) | Phosphatase | **1.000** |
| Ursolic acid | AKR1B10 (Aldose reductase-like) | Enzyme | **1.000** |
| Ursolic acid | PLA2G1B (Phospholipase A2) | Enzyme | **1.000** |

**Table S6.** PASS Online-predicted biological activity spectra for the four prioritized lead compounds from *A. decorticans* (Pa > 0.70 and Pa > Pi).

| **Biological activity** | **Amentoflavone** | **Kaempferol** | **Rosmarinic acid** | **Ursolic acid** | **Category** |
| --- | --- | --- | --- | --- | --- |
| Membrane integrity agonist | **0.969** | **0.974** | **0.956** | — | *Antimicrobial* |
| Membrane permeability inhibitor | **0.962** | **0.957** | 0.785 | — | *Antimicrobial* |
| HIF1A expression inhibitor | **0.973** | **0.969** | — | — | *Anticancer* |
| Kinase inhibitor | **0.964** | **0.959** | — | — | *Anticancer* |
| Antimutagenic | **0.952** | **0.948** | 0.722 | — | *Anticancer* |
| Peroxidase inhibitor | **0.948** | **0.956** | — | — | *Antioxidant* |
| HMOX1 expression enhancer | **0.953** | **0.945** | — | — | *Antioxidant* |
| Apoptosis agonist | **0.903** | 0.881 | — | 0.890 | *Anticancer* |
| TP53 expression enhancer | **0.939** | **0.931** | — | — | *Anticancer* |
| NADPH oxidase inhibitor | **0.918** | 0.889 | — | — | *Antioxidant* |
| MAP kinase stimulant | 0.897 | **0.917** | — | — | *Signalling* |
| Antineoplastic | 0.826 | 0.791 | 0.763 | 0.857 | *Anticancer* |
| JAK2 expression inhibitor | 0.701 | 0.745 | 0.745 | — | *Signalling* |
| Antioxidant | 0.879 | 0.856 | — | — | *Antioxidant* |
| Free radical scavenger | 0.780 | 0.771 | 0.745 | — | *Antioxidant* |
| MMP9 expression inhibitor | 0.702 | 0.738 | 0.730 | — | *Signalling* |
| Vasoprotector | 0.837 | 0.807 | — | — | *Vascular* |
| Cardioprotectant | 0.840 | 0.814 | — | — | *Vascular* |
| Antihypoxic | — | — | **0.921** | — | *Metabolic* |
| Antidiabetic | — | — | 0.799 | — | *Metabolic* |
| Hepatoprotectant | — | — | — | **0.961** | *Metabolic* |
| NF-κB stimulant | — | — | — | **0.927** | *Signalling* |
| Caspase 3 stimulant | — | — | — | **0.912** | *Anticancer* |
| Antiinflammatory | — | — | — | 0.864 | *Metabolic* |
| Hypolipemic | — | — | — | 0.885 | *Metabolic* |
| **Genotoxic †** | 0.852 | 0.843 | — | — | *Adverse* |
| **Toxic, vascular †** | 0.788 | 0.773 | — | — | *Adverse* |
